# Accurate Generation of Conformational Ensembles for Intrinsically Disordered Proteins with IDPFold

**DOI:** 10.1002/advs.202511636

**Published:** 2025-10-13

**Authors:** Junjie Zhu, Zhengxin Li, Zhuoqi Zheng, Bo Zhang, Bozitao Zhong, Jie Bai, Xiaokun Hong, Taifeng Wang, Ting Wei, Jianyi Yang, Hai‐Feng Chen

**Affiliations:** ^1^ State Key Laboratory of Microbial Metabolism Department of Bioinformatics and Biostatistics SJTU‐Yale Joint Center for Biostatistics National Experimental Teaching Center for Life Sciences and Biotechnology School of Life Sciences and Biotechnology Shanghai Jiao Tong University Shanghai 200240 China; ^2^ College of Biological Science and Engineering Fuzhou University Fuzhou Fujian 350116 China; ^3^ BioMap 10 Beilun Industrial Park, Yongteng North Road Beijing 100080 China; ^4^ MOE Frontiers Science Center for Nonlinear Expectations Research Center for Mathematics and Interdisciplinary Sciences Shandong University Qingdao 266237 China

**Keywords:** accurate generation, dynamical conformation ensemble, fine‐tuning, IDPFold, intrinsically disordered proteins

## Abstract

Intrinsically disordered proteins (IDPs) play pivotal roles in various biological functions whose dynamic structures are closely associated with many human diseases, including cancer, diabetes, and *Alzheimer disease*. Structural investigations of IDPs typically involve a combination of molecular dynamics (MD) simulations and experimental data to mitigate intrinsic biases in simulation methods. However, the high computational cost of these simulations and the limited availability of experimental data significantly restrict their applicability. Despite the recent advancements in structure prediction for structured proteins, understanding the conformational properties of IDPs remains challenging, partly due to the poor conservation of disordered protein sequences and the scarcity of experimental characterization. Here, IDPFold is introduced as a method capable of generating conformational ensembles for IDPs directly from their sequences using fine‐tuned diffusion models. IDPFold eliminates the reliance on multiple sequence alignments (MSA) or experimental data, offering a more detailed characterization of structural features in IDP ensembles. Evaluated across 27 IDP systems, IDPFold achieves Rg error of −0.06 and an RMSD of 0.65 ppm on Cα secondary chemical shifts with experimental values, significantly better than all existing generative deep learning approaches. IDPFold can be used to elucidate the sequence‐disorder‐function paradigm of IDPs.

## Graphical abstract

In brief: IDPFold predicts the conformational ensembles of intrinsically disordered proteins (IDPs) from their amino acid sequences. The atomic structures generated by IDPFold offer crucial insights into the intricate relationship between IDP structures and their diverse biological functions.2

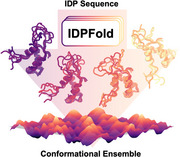

## 1 Introduction

Intrinsically disordered proteins (IDPs) constitute a category of proteins with unstable structures under physiological conditions.^[^ ^1^ ^]^ These proteins, accounting for over 40% of eukaryotic proteomes,^[^ ^2^, ^3^ ^]^ are involved in various biological functions including signal transduction, molecular recognition, and cell cycle regulation.^[^ ^4^, ^5^, ^6^ ^]^ IDPs are also closely associated with various significant diseases, such as cancer, Parkinson's disease, and acquired immunodeficiency syndrome (AIDS).^[^ ^7^, ^8^, ^9^ ^]^ Unlike structured proteins that possess one or a few stable conformations, IDPs exhibit transitions between multiple conformations with very low energy barriers, constantly fluctuating within a broad ensemble of structures under physiological conditions.^[^ ^10^, ^11^, ^12^ ^]^ Consequently, deciphering the conformational ensemble of IDPs poses a significant challenge for experimental methods such as X‐ray, cryo‐electron microscopy, and NMR.^[^ ^13^, ^14^, ^15^, ^16^ ^]^

While identifying disordered regions is well‐studied, with numerous computational tools, such as IUPred and IDP‐ELM, offering accurate and rapid prediction of disordered regions, the structural sampling of IDPs remains considerably more challenging.^[^ ^17^, ^18^ ^]^ Currently, molecular dynamics (MD) simulation is the most commonly used and effective tool for sampling conformational ensembles.^[^ ^19^, ^20^ ^]^ By iteratively sampling the target molecular system based on the first principles, an estimation of the conformational ensemble is obtained from simulation. MD simulations are broadly categorized into all‐atom and coarse‐grained (CG) simulations by their resolution.

All‐atom simulations, while offering detailed insights, often face substantial computational demands, making exhaustive sampling of conformational ensembles challenging. Additionally, commonly used all‐atom force fields for IDPs, such as ff03CMAP.^[^ ^21^ ^]^ a99SB‐disp,^[^ ^20^ ^]^ ESFF1,^[^ ^22^ ^]^ still exhibit considerable errors in estimating local and global properties of IDPs during simulations.^[^ ^23^, ^24^ ^]^ Conversely, CG simulations have demonstrated remarkable efficiency and accuracy in elucidating IDP dynamics. Sampling the equilibrium ensemble for a single‐chain IDP with residue‐based CG force fields like CALVADOS and Mpipi typically requires only a few minutes and is often accurate in estimating compaction of IDPs.^[^ ^25^, ^26^ ^]^ However, CG simulations lose details about local dynamics in proteins, providing only a smoothed free energy landscape.^[^ ^27^ ^]^

Experimental characterizations of IDPs can help in correlating all‐atom simulations, complementing missing details in CG simulations and developing simulation‐free sampling methods.^[^ ^28^, ^29^, ^30^ ^]^ However, the number of experimentally characterized IDPs remains limited. The Biological Magnetic Resonance Bank (BMRB) collected biological NMR data, which represent a primary resource for getting insight into local dynamics of proteins, especially IDPs.^[^ ^31^ ^]^ However, BMRB currently contains only 17071 entries, with only less than half possessing resolved structures. There is even fewer data regarding global dynamics, which is often featured by single‐molecule fluorescence spectroscopy (smFRET) and small‐angle X‐ray scattering (SAXS).^[^ ^32^ ^]^

On the other hand, numerous deep learning methods have been developed and widely applied in the field of structure prediction for structured proteins, such as AlphaFold2 and ESMFold.^[^ ^33^, ^34^ ^]^ Simultaneously, in protein design tasks, various generative models, such as RFdiffusion and Chroma, have been utilized for generating diverse protein backbones.^[^ ^35^, ^36^ ^]^ Therefore, it is natural to consider whether deep learning methods can be employed for rapid and accurate prediction of conformational ensembles of IDPs.

However, the structures associated with IDPs are currently very sparse. The PDB database contains > 220000 structural entries,^[^ ^37^ ^]^ while the Protein Ensemble Database only includes 553 protein ensemble data.^[^ ^38^ ^]^ A critical resource for studying IDPs is MobiDB, which aggregates disorder annotations for over 245 million proteins, but high‐quality structures, let alone conformational ensembles, for these disordered proteins remain scarce.^[^ ^39^, ^40^ ^]^ What compounds this challenge is the frequent lack of high‐quality multiple sequence alignment (MSA) data for IDPs. MSAs have been proven effective only in functional studies concerning folded or bound states of IDPs, while providing little assistance in predicting more disordered conformations.^[^ ^41^ ^]^ These significant data limitations make it highly daunting to use deep learning for robustly predicting the conformational ensembles of IDPs. Although Janson et al. have previously worked on IDP conformation generation and proposed idpGAN for predicting IDP conformational ensembles, they mainly forced on generating coarse‐grained IDP conformations and often suffer from over‐sampling, leaving a gap to coarse‐grained or even all‐atom MD simulations.^[^ ^42^, ^43^ ^]^

We introduce IDPFold here for predicting IDP dynamics directly from sequences based on a generative deep learning model. IDPFold utilized a protein language model to extract sequence information and further fed it into a structure generation module, enabling MSA‐free conformation generation. To address the issue of insufficient data, we employed a hybrid dataset comprising crystal structures, NMR structures, and MD trajectories to train IDPFold. The experimental structures enable the model to learn basic protein characteristics, while MD trajectories provide sufficient IDP structural data, ensuring accurate sampling on IDP systems. IDPFold generates IDP conformational ensembles at the backbone level and is in better agreement with experimental observations than other state‐of‐the‐art methods. IDPFold is able to sample both structured and disordered states of proteins, providing insights for studying the correlation between structures and functions of IDPs.

## 2 Results

**Figure 1:**
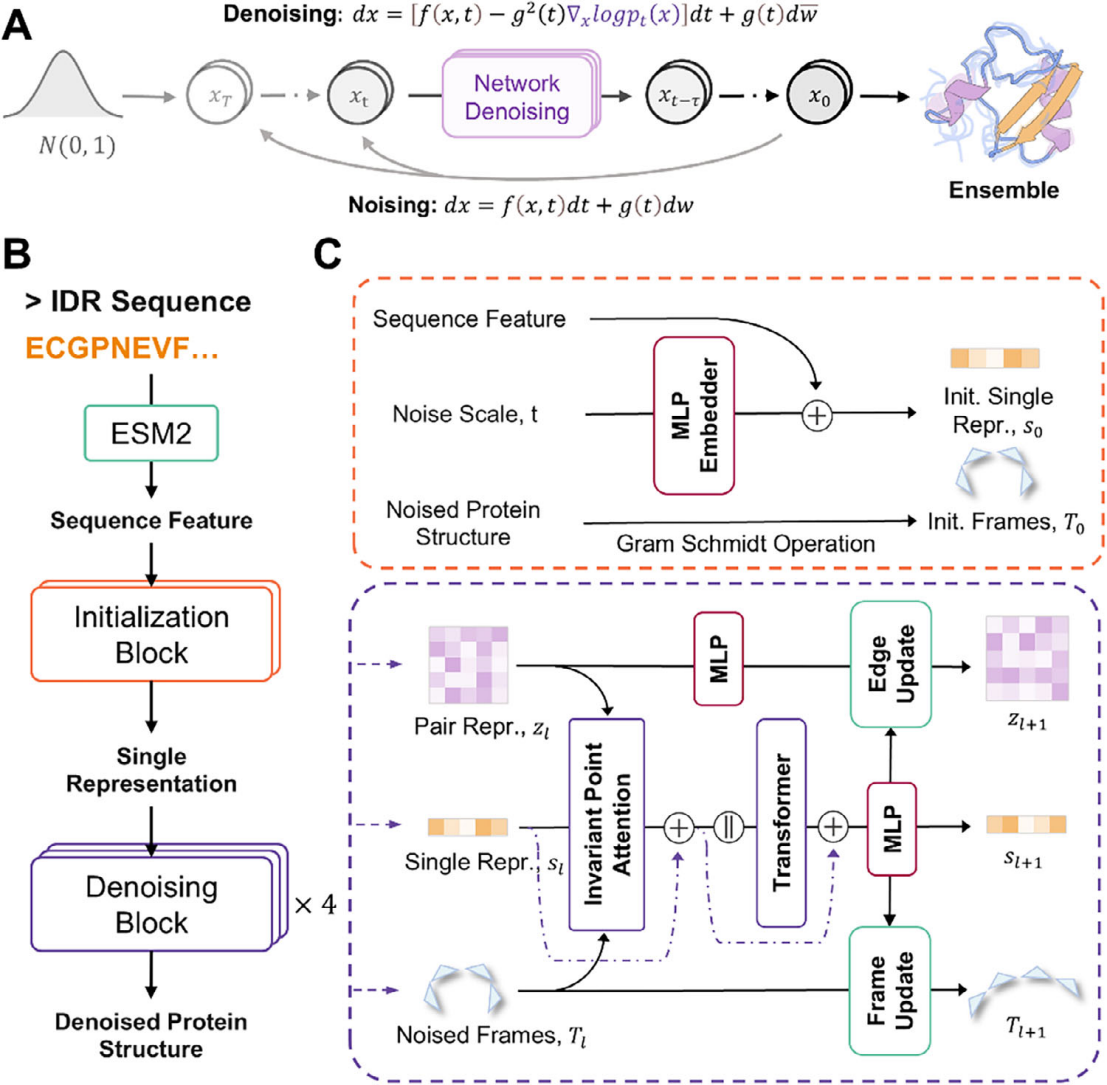
Architecture of IDPFold. A) Diffusion process for generating protein ensembles. B) Structure of the denoising network. C) Detailed architecture of Initialization Block and Denoising Blocks.

IDPFold employs a conditional diffusion model framework for generating protein conformational ensembles from sequences (**Figure** **1**A). This framework involves a forward diffusion process where the noise is gradually added to real protein structures, and a reverse diffusion process where a deep learning network is used for denoising. By integrating specific protein sequence features into the model during the reverse diffusion process, we can generate conformational ensembles for specific proteins using this architecture. The denoising network takes as input the sequence features extracted by ESM2 and consists of an initialization block and four denoising blocks (Figure 1B). The initialization module integrates the sequence features, noise scale, and noise structure, while denoising modules combine Invariant Point Attention (IPA) with traditional Transformers to capture the chain‐like structure within proteins and the rotational/translational state of each residue (Figure 1C). For more details, please refer to the Materials and Methods section.

### 2.1 IDPFold Reproduces Global Features of IDPs

**Figure 2:**
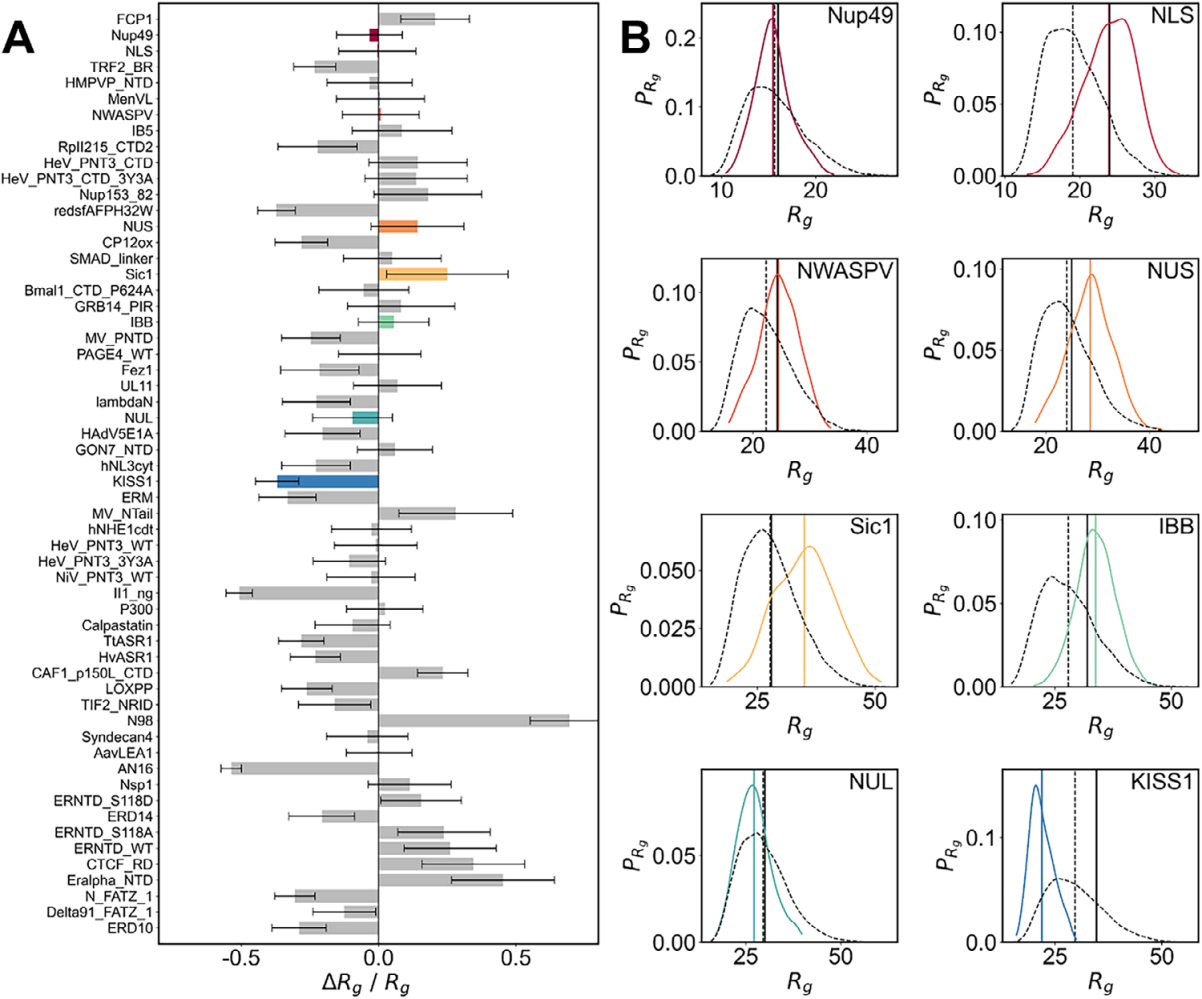
Comparison between the generated ensembles of IDPFold and coarse‐grained MD simulation with CALVADOS 2. A) Relative error between IDPFold predicted and experimental radii of gyration on the test set of CALVADOS 2. B) Rg distributions of IDPFold‐generated ensembles (colored) and CALVADOS simulations (black dashed). The unit of Rg is angstrom (Å). Experimental values are plotted as solid black lines. 300 conformations are sampled by IDPFold or extracted from the MD trajectory for each system.

We first evaluated the IDPFold‐predicted ensembles at a coarse‐grained level, primarily focusing on the global characteristic of the predicted ensembles, specifically the radius of gyration (Rg). Rg is a critical physical quantity that reflects the global characteristic of IDPs, with its magnitude positively correlating with overall protein looseness, making it an empirical measure to describe and distinguish IDPs from structured proteins.^[^ ^44^, ^45^ ^]^ We first referred to the evaluation by Tesei et al. on the coarse‐grained force field CALVADOS 2 by calculating the Rg error of the IDPFold‐predicted ensembles on their test set. After removing sequences presented in the training set, this test set contained 58 IDP systems. Among these proteins, the average Rg error of the IDPFold‐predicted ensembles was − 6%. (**Figure** **2**A). We further examined the Rg distribution of predicted ensembles for each system and compared it with coarse‐grained trajectories. The results demonstrated that the ranges of Rg distribution of IDPFold‐predicted ensembles closely matched that of the coarse‐grained simulations for most systems (Figure 2B; Figure S1, Supporting Information), though there are some systems, such as PaaA2 and drkN‐SH3, with significant errors.

### 2.2 IDPFold Captures the Global Distribution of IDP Ensembles

To further assess the robustness of IDPFold, we collected 27 IDP systems that possessed rich experimental observation data and were not present in the training set (Tables S1 and S2, Supporting Information). We compared the IDPFold‐predicted ensembles with the simulation results from CALVADOS 2 and the predictions from the coarse‐grained deep learning method idpGAN on these systems.^[^ ^42^, ^46^ ^]^ The result showcased that the average Rg error of the IDPFold‐predicted ensembles on this test set (ε_*Rg*_ =  Δ*R_g_*/ *R_g_* =   − 0.06) was significantly smaller than that of idpGAN (ε_*Rg*_ =   − 0.12, with a paired t‐test p‐value of 0.02). The Rg error of the simulation trajectories was 0.02, which is better in absolute terms than both deep learning methods, while without a significant difference with IDPFold (a paired *t*‐test p‐value of 0.12). Compared to coarse‐grained simulations, IDPFold tends to slightly underestimate Rg for IDPs, particularly for longer proteins. This is partly due to the high conformational space complexity of long IDPs, making it more challenging to model their conformational ensembles. Although the fine‐tuned version of IDPFold corrected the disorder tendency in the generated conformations to a certain extent, it still produced partly structured estimation for large systems and resulted in overestimated compaction. Overall, IDPFold tends to have a lower proportion of highly extended conformations in the predicted ensembles for large systems. This opens a future direction toward the improvements on the accuracy of global characteristic estimates by reweighting to increase the proportion of these extended conformations in the ensembles.

**Figure 3:**
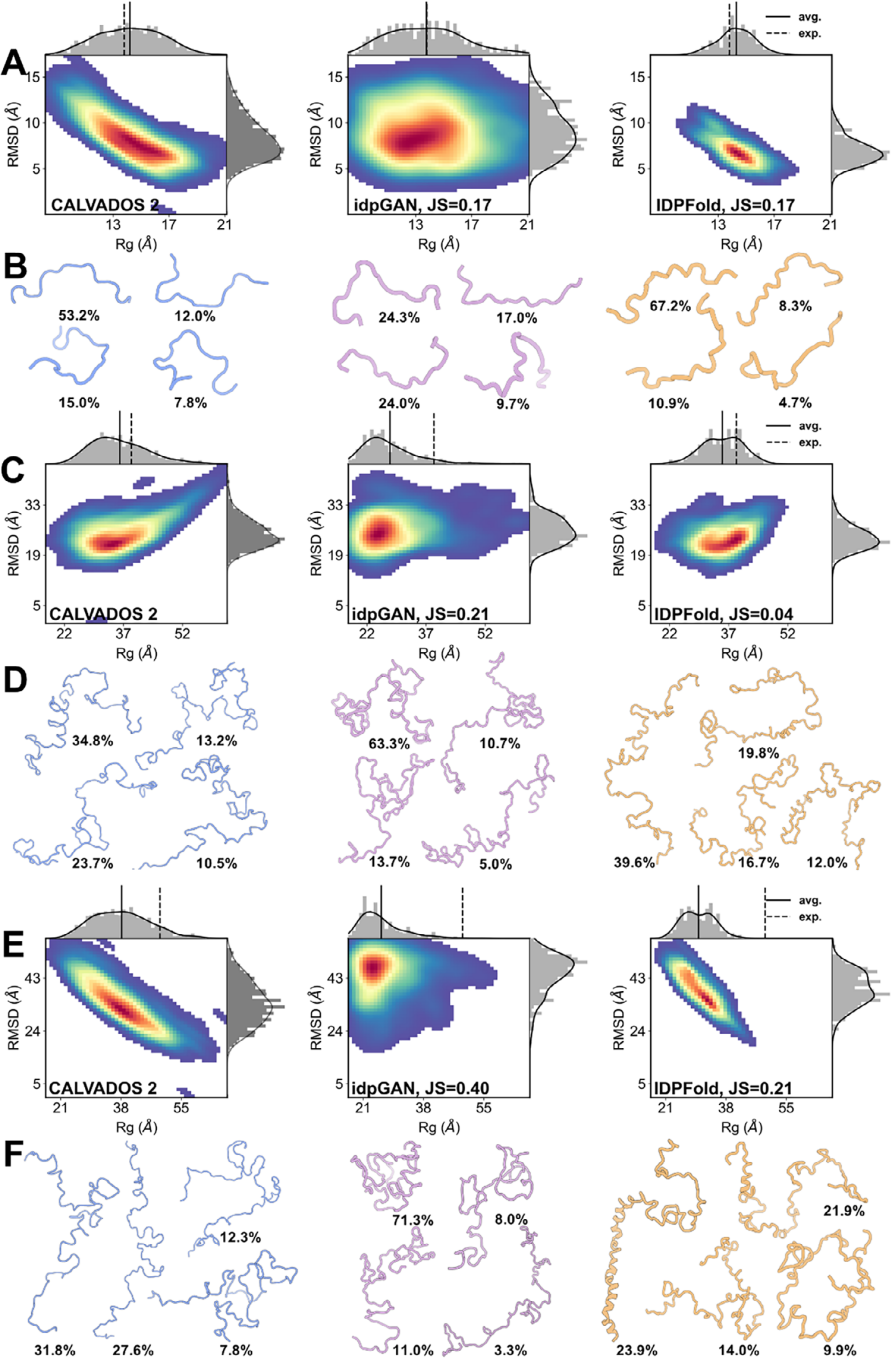
Comparing the generated ensembles of IDPFold and the coarse‐grained deep learning method idpGAN. A, B, Rg‐RMSD distribution A) and cluster centers B) of predicted ensembles on Histatin5. C,D) Rg‐RMSD distribution (C) and cluster centers (D) of predicted ensembles on Human Calpastatin. E,F) Rg‐RMSD distribution (E) and cluster centers (F) of predicted ensembles on β Synuclein. 300 conformations are sampled for each system.

We next sought to observe the ensemble distributions and main conformations generated by the three methods on specific cases. Here, we calculated the Rg‐RMSD distribution of each ensemble using the initial conformations from coarse‐grained simulations as a reference. The results showed that idpGAN exhibited over‐sampling across all test systems and had significant deviations in estimating the main conformations. As a comparison, the ensemble distribution estimated by IDPFold was closer to coarse‐grained trajectories, with its estimation of the free energy well positions more accurate than that of idpGAN (**Figure** **3**A,C,E). Although the sampling range of IDPFold was smaller than that of coarse‐grained trajectories, its estimation of the ensemble's Boltzmann distribution was more precise than that of idpGAN.

From the clustering results, the main conformations and their proportions sampled by IDPFold across the three systems were closer to the simulation results than idpGAN. In the Histatin5 protein, both IDPFold‐predicted ensembles and the simulation trajectories had an extended conformation as the main structure, whereas the main conformation predicted by idpGAN was in a semi‐folded state (Figure 3B). In the two larger systems, Human Calpastatin and β Synuclein, the issues with idpGAN were even more pronounced, as it predicted collapsed main conformations, which led to a severe underestimation of the average Rg of these two proteins (Figure 3D,F). This indicates that idpGAN's estimation of the ensemble distribution is inaccurate. On the other hand, IDPFold‐predicted ensembles more accurately captured the shapes of the main conformations. Although IDPFold underestimated the Rg on β Synuclein due to overestimating the helical tendency in the structure, its estimates of the proportions of extended and folded conformations were closer to the simulation results.

### 2.3 IDPFold Predicts IDP Ensembles Comparable to all‐Atom MD Simulations

A major advantage of IDPFold compared to the aforementioned coarse‐grained simulations is its capability to generate protein at the backbone level, allowing us to better understand the dynamic properties of proteins at higher precision. Thus, we conducted all‐atom MD simulations on 27 IDP systems in the test set to assess the quality of the local features in the IDPFold‐predicted ensembles. We used the ESFF1 force field and solvent model OPC3‐B, which are specifically parameterized for IDPs.^[^ ^22^, ^24^ ^]^ To demonstrate the convergence of our simulation, we performed three independent simulations on 10 of these proteins, recording the distributions and mean values of experimentally observed physical quantities across the parallel trajectories. The results of the convergence analysis are shown in Figure S2 (Supporting Information). Subsequently, we compared the IDPFold‐predicted ensembles with the results from the all‐atom MD simulations.

We examined the distributions of bond lengths, bond angles, and dihedral angles predicted by IDPFold. In terms of bond length and bond angle distributions, the ensemble predicted by IDPFold closely resembles those observed in the MD trajectories, indicating that the model has effectively learned the arrangement of side chains in protein residues, accurately predicting the distances and arrangements between adjacent atoms (Figure S3A, Supporting Information). Additionally, the distribution of Ω angles in the conformations generated by the model resemble those observed in MD trajectories, showcasing a peak ≈180°, suggesting that the local peptide bond plane conformations generated by the model are stable and align with general protein characteristics (Figure S3B, Supporting Information). Moreover, the Ramachandran plot of φ − ψ angles show that the backbone dihedral angles of generated conformations predominantly fall within reasonable regions. The density estimation across various regions also closely approximates those observed in MD trajectories (Figure S3C–E, Supporting Information). Specifically, with IDPFold fine‐tuned on IDRome data, there is a notable improvement on the probability of the ppII region in the upper left corner compared to the untuned version, showing a distribution closer to MD trajectories. This indicates that the fine‐tuning enables the model to generate more disordered structures, capturing the intrinsic biases of target proteins more precisely.

**Figure 4:**
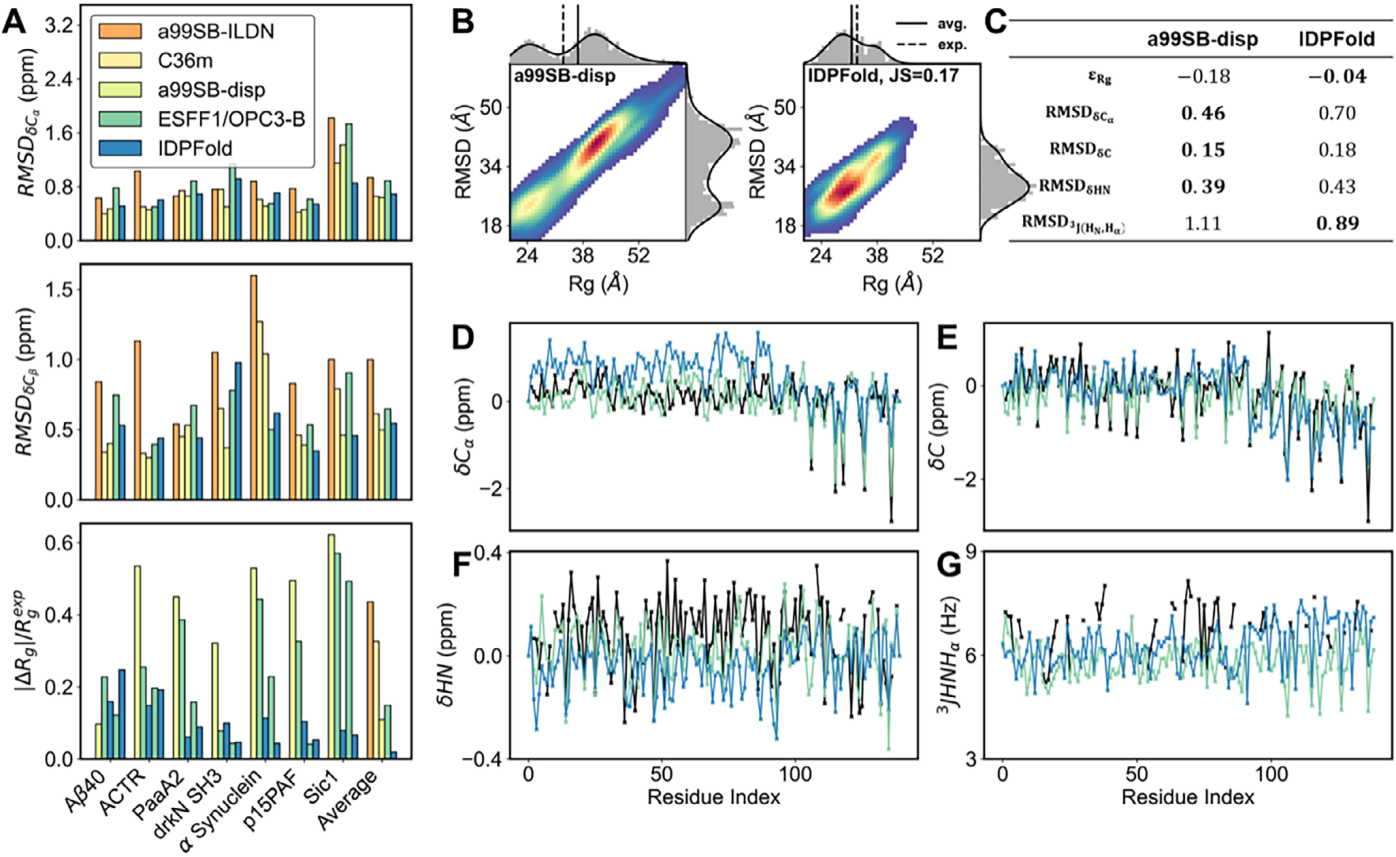
Comparison between the performance of IDPFold and that of all‐atom MD simulations with different force fields. A) Accuracy of IDPFold in estimating local features (*C*_α_, *C*_β_ chemical shifts) and global feature Rg compared to MD simulations. B) Rg‐RMSD distribution of IDPFold‐generated ensemble compared to MD simulation with a99SB‐disp on α Synuclein. C) Errors between generated ensembles and experimental observations for a99SB‐disp and IDPFold on α Synuclein. D–G) Local Features including *C*_α_ chemical shifts (D), *C* chemical shifts (E), *HN* chemical shifts (F) and 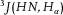 (G) of IDPFold generated ensembles and MD simulation with a99SB‐disp on α Synuclein. 300 conformations are sampled for each system.

While bond fluctuations only represent local features that converge fast in simulation, global features hardly converge within one microsecond. Therefore, we additionally collected long trajectories used by Robustelli et al. in testing the a99SB‐disp force field, along with other force field trajectories for comparison.^[^ ^20^ ^]^ We analyzed the differences in performance between IDPFold and four all‐atom molecular dynamics simulations on seven proteins that overlapped with our test set. We primarily focused on the Rg errors and the RMSDs of *C*_α_ and *C*_β_ secondary chemical shifts (**Figure** **4**A; Figure S3F–H, Supporting Information).

In terms of chemical shift accuracy, the error in the IDPFold‐predicted ensembles reached a level comparable to a99SB‐disp, surpassing traditional force fields such as a99SB‐ILDN and CHARMM36m.^[^ ^19^, ^47^ ^]^ Furthermore, IDPFold demonstrated significantly better performance in Rg error compared to all four force fields, indicating that IDPFold accurately captures the global characteristics of IDPs while achieving a comparable level of precision in local structure prediction as current state‐of‐the‐art force fields. Using α Synuclein protein as an example, we illustrated the differences between the ensembles sampled by IDPFold and the 70‐microsecond MD trajectories from the a99SB‐disp force field. We calculated the *R_g_*‐*R*_*e*2*e*_ distributions from both methods and found that while a99SB‐disp sampled a wider range of conformations and captured more extended states, while IDPFold predominantly sampled more compact conformations (Figure 4B). In terms of Cα chemical shifts, we observed an overestimation of helical propensity in the N‐terminal region of α‐Synuclein (Figure 4C,D). A considerable β‐sheet population was also noted between residues 35–50 in drkN‐SH3. This suggests that IDPFold might more readily capture the folded states of these IDPs than their unfolded states, likely due to the prevalence of similar folded structures in the training set. It should also be noted that the AlphaFold and ESMFold predicted structures of alpha‐synuclein are also very helical and look more like the membrane‐bound synuclein structure. This suggests that all of these models retain some bias in the sequence model that does not reflect the solution state. For C and HN secondary chemical shifts, RMSDs of IDPFold were slightly larger than those of a99SB‐disp, while in 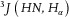 scalar coupling, IDPFold slightly outperformed a99SB‐disp. Overall, we conclude that IDPFold's characterization of IDP local features is on par with traditional force fields, and its sampling of α Synuclein takes ≈20 min, significantly faster than the hundreds of hours required by traditional MD simulations. This demonstrates the power of IDPFold to serve as a complementary tool to traditional force field sampling, which might pave the way for the exploration of macromolecular systems on a considerable time scale.

### 2.4 IDPFold Outperforms Existing Deep Learning‐Based Methods

Lastly, we compared IDPFold with existing deep learning‐based methods on the test set, using experimental observations as the primary references to establish a benchmark for ensemble prediction methods (**Table** **1**). This benchmark includes three coarse‐grained methods and four methods with backbone‐level or higher accuracy, in which AlphaFlow of the best performance (AF‐PDB‐base) is presented (Table S3, Supporting Information). In terms of global characteristics like Rg, IDPFold was slightly outperformed by the coarse‐grained force field CALVADOS 2 (Figures S4–S9, Supporting Information). In some cases, the ensembles generated by IDPFold deviate significantly from the experimental Rg, primarily due to the underestimation of the proportion of highly disordered conformers. The conformations simulated by CALVADOS 2 are in a fully disordered state. It failed to sample folded or partial‐folded states of IDPs, even if it yielded accurate Rg estimates. In contrast, IDPFold was capable of sampling these structures and achieved the best performance across most local metrics among backbone‐level methods (Figures S10–S15, Supporting Information). We also evaluated the validity of the conformations generated by various deep learning methods (as defined in the materials and methods section). The results showed that all existing deep learning methods generated conformations with high validity. However, we observed a slight decrease in the validity of IDPFold‐generated ensembles after fine‐tuning, likely due to the greater local structural fluctuations in the trajectory data used for fine‐tuning compared to experimental structures, which led to more pronounced local fluctuations in the generated conformations (Figure S16, Supporting Information). Overall, IDPFold‐predicted ensembles outperformed existing deep learning methods in all experimental observations and were comparable to or even better than traditional MD simulations.

**Table 1:** Benchmark on IDPFold and other methods. Bold values denote the best.

Methods	Validity	ε_*Rg*_	$RMSD_{\delta C_{\alpha }}\left(ppm\right)$	$RMSD_{\delta C_{\beta }}\left(ppm\right)$	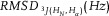	$RMSD_{RDC_{NH}}\left(Hz\right)$
CALVADOS 2^[^ ^48^ ^]^	–	0.02	–	–	–	–
idpGAN^[^ ^42^ ^]^	0.91	− 0.12	–	–	–	–
idpSAM^[^ ^49^ ^]^	0.95	− 0.55	–	–	–	–
STARLING^[^ ^50^ ^]^	0.93	0.06	–	–	–	–
a99SB‐disp^[^ ^20^ ^]^ (7 systems)	–	0.11	**0.65**	**0.49**	**0.72**	4.30
MD 3*1µs ESFF1+OPC3‐B	–	− 0.19	0.81	0.64	0.96	3.37
bAIes^[^ ^51^ ^]^	0.92	− 0.10	0.66	0.67	0.89	3.83
AF‐cluster^[^ ^52^ ^]^	**0.99**	− 0.12	0.74	1.30	1.81	3.84
AlphaFlow^[^ ^53^ ^]^	0.97	− 0.24	0.71	1.16	1.26	4.02
BioEmu^[^ ^54^ ^]^	0.97	− 0.18	0.88	1.28	1.05	5.21
IDPFold	0.95	− 0.06	**0.65**	0.53	1.01	**3**.**27**

## 3 Discussion

In this study, we developed IDPFold, a deep learning‐based tool for generating IDP conformational ensembles. IDPFold adopts a conditional diffusion model architecture to perform end‐to‐end protein conformational generation, integrating protein language model for sequence feature extraction and DenoisingIPA module for conformation denoising. Through a two‐stage training strategy on experimental data and MD trajectories of IDPs, respectively, IDPFold efficiently and accurately samples IDP conformational ensembles. Furthermore, we have established a benchmark of conformation sampling methods on 27 protein systems that contain IDRs. IDPFold precisely captures the overall compactness and local secondary structural features of IDPs, with predicted ensembles exhibiting features with values closer to experimental observations compared to both existing MD‐based and deep‐learning methods. Additionally, IDPFold can sample conformations of both structured and disordered states in proteins, demonstrating a wide sampling range and high efficiency in ensemble sampling. This capability provides important insights for studying the conformational changes and functions of IDPs.

IDPFold achieves accuracies comparable to or even higher than traditional MD simulations in estimating IDP conformational ensembles, with its sampling process not restricted by energy barriers. This is not only essential for studying IDP conformations but also holds tremendous promise for dynamic proteins like allosteric proteins and enzymes, which undergo large‐scale conformational changes during functional processes. Although this work focused on training the model for IDPs where direct prediction performance on allosteric protein ensembles might not be optimal, the methodology would likely to be transferred on diverse categories of proteins, enabling more accurate estimations of conformational transitions in these functionally important proteins (Figure S17, and Movie S1, Supporting Information).

Although IDPFold demonstrates robustness and precision in predicting conformational ensembles, its inference time is relatively long compared to some of the existing deep learning methods, with an average time of ≈20 min to sample an entire system. While this speed is much faster than conventional MD simulations, there is room for further improvement in model efficiency. To achieve higher sampling accuracy and more precisely protein backbone features, we employed a more complex network architecture and longer diffusion steps, which might well sacrifice sampling efficiency. To enhance the inference efficiency of the model, optimizations can be explored, such as reducing the number of diffusion steps in the diffusion model and optimizing transformer components by replacing them with architectures that are more efficient in terms of time and space.^[^ ^50^ ^]^ Additionally, our current study trained and tested IDPFold exclusively on single‐chain proteins, without assessing its performance on protein complexes or multi‐chain assemblies. Future work will therefore aim to extend deep learning‐based structure generation to these more complex systems, enabling efficient sampling and mechanistic interpretation of dynamic intermolecular interactions.^[^ ^55^ ^]^ Despite the low average Rg error, IDPFold shows significant error for certain systems (Figure 2A), primarily resulting from inaccurate estimation of the proportion of folded states, such as α‐helix in PaaA2 and β‐sheet in drkN‐SH3. This contrasts with CG simulations, which could accurately estimate Rg and often fail to sample stable folded structures. This suggests a critical trade‐off between accurately estimating global features and sampling stable local structures. A potential solution for future work could involve a hybrid modeling approach where a CG component focuses on global dynamics and an all‐atom component handles local structural details, allowing for accurate modeling of both aspects.

It should also be noted that in this work, we primarily used a limited set of experimental features for evaluating predicted conformational ensembles. These experimental characterizations, alongside insights from other computational approaches, also offer significant potential for refining the predicted ensembles. Several recent works have demonstrated that the static structure predicted by AlphaFold2 can serve as a vital reference for Bayesian inference of IDP ensembles.^[^ ^56^, ^57^ ^]^ Similarly, incorporating experimental features like Rg into diffusion models has shown promise for more accurate ensemble predictions.^[^ ^55^ ^]^ Furthermore, CG parameters are proven effective in capturing dynamic interactions among IDRs.^[^ ^58^ ^]^ IDPFold provides an end‐to‐end pipeline for predicting IDP conformational ensembles solely from their sequences. Moving forward, further correlations and refinements of these predicted ensembles can be achieved by integrating additional experimental features and leveraging more sophisticated computational references.

Recent advances highlight the potential for sequence‐based prediction of complex molecular interactions. Garrett M. Ginell et al. introduced FINCHES, a framework that repurposes chemical potentials from CG force fields to directly estimate IDR‐mediated attractive and repulsive interactions, generate intermolecular interaction maps, and predict homotypic phase diagrams from sequence alone.^[^ ^58^ ^]^ Meanwhile, Sören von Bülow et al. have combined coarse‐grained molecular dynamics with active learning to train ML models that accurately predict free energies and saturation concentrations for IDR phase separation, applying their model to ≈27000 human IDRs and identifying ≈5 % prone to homotypic phase separation.^[^ ^59^ ^]^ These works exemplify powerful, sequence‐based approaches to predict IDR‐associated intermolecular interactions. However, their reliance on CG force fields inherently limits resolution to non‐bonded interaction patterns and meanfield behaviors. To fully capture the detailed atomic determinants of protein–protein interactions, it will be critical to extend conformational generative models or integrate these interaction predictors into all‐atom resolution frameworks.

Conformational ensemble prediction represents a significant frontier in protein structure research following static structure prediction. Accurate characterization of protein conformational ensembles can help us understand the dynamics of proteins and crucial conformational changes they undergo when binding to substrates or undergoing biochemical reactions under physiological conditions. Although deep learning‐based tools are not yet widely adopted and most dynamic analyses still rely on MD simulations, our work suggests that properties predicted using deep learning methods might offer higher accuracy than MD simulations, potentially serving as complementary or even alternative approaches to MD simulations. However, current deep learning‐based conformational ensemble prediction methods still face several challenges, such as inaccurate estimation of conformational free energies, less robustness compared to traditional methods, and the inability to capture temporal autocorrelation between conformations (i.e., inability to capture dynamic features).

Several studies have indicated that the single‐point energy estimation of molecular force fields can be used to train diffusion models, allowing models to learn more comprehensively about conformational space in cases where structural data are scarce.^[^ ^60^, ^61^ ^]^ This training approach, which utilizes force field energies rather than structural data, enables more accurate capture of conformational space distribution and greater robustness. However, this training strategy also makes the model sensitive to empirical parameters of the force field, demanding high precision in molecular force field accuracy. Additionally, there are works based on flow models or score‐matching models aiming to fit the dynamic characteristics of MD trajectories, which might be solutions to the current inability of generative models to capture temporal autocorrelation between conformations.^[^ ^62^ ^]^

Apart from the challenges of sparse training data and limited model representation capabilities, there is another significant issue in conformational ensemble prediction tasks: the scarcity and lack of uniformity in evaluation metrics. While we collected a set of experimental observations as a gold standard for this study, a vast number of protein systems lack experimental annotations, making it challenging to assess the generative performance of computational models on these systems. Therefore, we believe that constructing confidence metrics for conformational ensemble generation akin to pLDDT for protein structure prediction is also promising for the evaluation and improvement of protein conformational ensemble models.

## 4 Experimental Section

### Datasets

The data for training IDPFold consists of three components: high‐quality crystal structures collected from the PDB dataset, structures derived from NMR and molecular dynamics (MD) simulation dataset of IDPs obtained through extensive back‐mapping and energy minimization on coarse‐grained trajectories.

For crystal structures, we referenced trRosetta and gathered a total of 15051 X‐ray resolved structures with resolutions ≤ 2.5 Å and sequence redundancy ≤ 30%.^[^ ^63^ ^]^ These structures provide fundamental protein characteristics for the model, such as bond lengths, bond angle distributions, and chirality of *C*_α_. However, crystal structures typically depict stable conformations of proteins, i.e., structured conformations. Training the model solely on crystal structures would significantly underestimate the disorder tendency of IDPs and result in low diversity in conformation generation for individual protein sequences. Therefore, additionally, 12 339 NMR resolved protein conformational ensembles were collected from the PDB. These NMR entries were filtered based on 30% sequence similarity both internally and against crystal structures, resulting in 539 systems comprising a total of 10 454 structures. By blending crystal structures with NMR ensembles, we obtained a combined total of 25 495 experimental structures for the initial phase of IDPFold training. These structures were well‐defined and exhibited a higher structured tendency, ensuring that the model captures the local physical characteristics of proteins effectively.

For the IDP trajectory data, we obtained large‐scale coarse‐grained simulation data from IDRome that recorded most IDRs in the human proteome.^[^ ^46^ ^]^ These coarse‐grained simulations accurately capture global features of IDPs (and IDRs), such as the average radius of gyration (Rg) of conformational ensembles, but they only retain *C*_α_ atoms, whose resolutions fail to meet the requirement for model training. Therefore, we selected all systems with lengths exceeding 256 residues, totaling 3880 systems. Choosing larger systems is primarily because these long‐disordered segments encompass most sequence and structural characteristics found in smaller systems, and training the model on larger systems can help enhance its generalization ability. For these systems, pdbfixer was first used to restore structures from coarse‐grained to all‐atom, and then performed 100 steps of energy minimization with ff14SB^[^ ^64^ ^]^ force field were performed for all protein structures. A total of 77 600 optimized all‐atom conformations was used for the second phase of IDPFold training, these conformations globally exhibit more disordered and could aid the model in learning distinctive conformational features of natural IDPs.

Furthermore, data used to assess the performance of IDPFold generation included 27 IDP systems that are fully described by experiments and are not present in the training set. All‐atom MD simulations of 1µ*s* were conducted for these systems using the IDP‐specific force field ESFF1 and solvent model OPC3‐B.^[^ ^22^, ^65^ ^]^ These all‐atom simulation trajectories described the dynamics and thermodynamic characteristics of IDPs, which were suitable for evaluating the quality of IDPFold‐generated conformational ensembles.

### Formulation of IDPFold—Diffusion Modeling on Protein Structure

To enable IDPFold to capture the Boltzmann distribution of protein conformations at the equilibrium state, Score‐Based Generative Modeling (SGM) was employed to learn the probability distribution from protein structure data. SGM could be represented by a diffusion process $x_{t}\in R^{n}$ defined by a stochastic differential equation (SDE).^[^ ^66^ ^]^ The forward diffusion process was characterized by the following equation:

(1) $$dx=f\left(x,t\right)dt+g\left(t\right)dw$$

where t∈[0,T] was a continuous index and $w\in R^{n}$ was the standard Wiener process (a.k.a., Brownian motion). $f\left(x,t\right)\in R^{n}$ was a vector‐valued function called the drift coefficient, and g(t)∈R was a scalar function called the diffusion coefficient. Then, the corresponding backward diffusion process, or denoising process, could also be defined by SDE^[^ ^67^ ^]^:

(2) $$dx=\left[f\left(x,t\right)-g^{2}\left(t\right)\nabla_{x}logp_{t}\left(x\right)\right]\;dt+g\left(t\right)d\bar{w}$$

where $d\bar{w}$ was a standard Wiener process as continuous time *t* flows backward from *T* to 0, and *dt* was an infinitesimal negative time step. *x*_*t*  =  0_ here represents ground truth data, or the protein conformations, while *x*_*t*  =  *T*_ was sampled from a Gaussian distribution. Therefore, by solving the backward SDE process, diverse protein conformations that obey Boltzmann distribution can be sampled from a Gaussian distribution. In Equation (2), each term except the score of *x*, ∇_*x*_ *logp_t_* (*x*), is solvable. Therefore, only a score‐matching network was required to fit ∇_*x*_ *logp_t_* (*x*) at each time step to achieve the purpose of generating conformations.

Based on the above‐mentioned standard formulation of SGM, It was further required that the entire conformation generation process should be SE(3)‐equivariant, i.e., diffusion process and network transformation were not sensitive to global rotation and translation of protein structures. SE(3)‐equivariance can be described by the following equation:

(3) $$F\circ \rho \;\left(x\right)=\rho \circ F\left(x\right)$$

where F denotes data transformations like network prediction and diffusion process, ρ represents global rotation and translation. Typically, a protein conformation *x* was characterized by Cartesian coordinates $c_{i}\in R^{3},1\leq i\leq N$, where *N* denotes the number of atoms. However, data transformations on Cartesian coordinates were computationally intensive and did not easily satisfy SE(3)‐equivariance. Therefore, backbone frame parametrization was adopted to represent the protein conformation *x* as $T_{j}≔\left[R_{j},v_{j}\right],1\leq j\leq n$, where *n* denotes the number of residues. Each backbone frame includes a 3 × 3 rotation matrix $R_{j}\in SO\left(3\right)$ and a translation vector $v_{j}\in R^{3}$. A frame *T_j_* can represent the Euclidean transformation for each atom in the residue *j* from local coordinates *c_local_* to global coordinates *c_global_* as $c_{global}=T_{i}\;\circ c_{local}≔R_{i}\;c_{local}+v_{i}$. Following the approach described in FrameDiff,^[^ ^61^ ^]^ the rotation matrix *R_j_* on the *SO*(3) manifold and the translation vector *v_j_* in $R^{3}$ are independently handled during the diffusion process, formulated as follows:

(4) $$\begin{array}{c} d\;T_{t}=\left[0,-\frac{1}{2}\beta \left(t\right)Pv_{t}\right]\;dt+\left[\sqrt{\frac{d}{dt}\sigma^{2}\left(t\right)}dw^{\left(SO\left(3\right)\right)},\sqrt{\beta \left(t\right)}Pdw^{\left(R^{3}\right)}\right] \end{array}$$

where β(*t*) and σ(*t*) control the scale of noise during the diffusion process, $w^{M}$ denotes Brownian motion defined on a manifold M, and $P:\;R^{3n}\to R^{3n}$ was used for removing the center of mass. During the forward diffusion or noising process, the addition of noise on rotation matrices was determined by the noise kernel *p*_*t*|0_(*R_t_* |*R*_0_), which was obtained from an isotropic Gaussian distribution on the *SO*(3) manifold. This distribution was formulated as:

(5) $$\begin{array}{ccc} IG_{SO\left(3\right)}\;\left(R_{t};R_{0},\sigma^{2}\right) & = & f\left(\omega_{t|0}\right)≔\frac{1-\cos\left(\omega_{t|0}\right)}{\pi }\\ & & \sum_{l=0}^{\in }fty\;\left(2l+1\right)e^{-l\left(l+1\right)\sigma^{2}\frac{\sin\left(\left(l+0.5\right)\omega_{t|0}\right)}{\sin\left(0.5\omega_{t|0}\right)}} \end{array}$$

Here, $\omega_{t|0}=Axis\_angle\left(R_{0}^{T}R_{t}\right)$ was the axis‐angle transformed representation of the composed rotation matrix $R_{0}^{T}R_{t}$. As for the translation vector, its noise addition process follows an Ornstein‐Uhlenbeck process, also known as VP‐SDE. The noise kernel for the translation vector was relatively straightforward, converging ultimately to N(0,I) as shown in the following equation.

(6) $$p_{t|0}\;\left(v_{t}|v_{0}\right)=N\left(v_{t};v_{0}e^{-\frac{1}{2}\int_{0}^{t}\beta \left(s\right)ds},I-Ie^{\int_{0}^{t}\beta \left(s\right)ds}\right)$$

### Formulation of IDPFold—Network Design and Training

To achieve the goal of predicting conformational ensembles from sequence, we devised a sequence‐conditioned score‐matching model $s_{\theta }\left(x_{t},x,seq\right)$ for denoising processes. As the diffusion process is designed to be strictly SE(3)‐equivariant, network transformations should evidently preserve this property. Therefore, a variant of the structure module from AlphaFold2 (Figure 1A) was adopted to update backbone frames.^[^ ^33^, ^43^ ^]^ Here, the Invariant Point Attention (IPA) mechanism was employed to capture interactions and relationships between nearby residues, followed by a Transformer to learn global features and long‐range interactions. This architecture has been demonstrated in previous research to promote training and the generation of high‐quality protein conformations. The aforementioned network design requires three inputs for each layer: a 1D vector representation *s_l_*, pairwise feature representation *z_l_*, and the set of rotation and translation updates *T_l_*. ESM2‐650M was utilized to extract protein sequence features, concatenated with residue position encoding and time encoding represented by trigonometric functions, to form the initial 1D vector representation *s*_0_. Pairwise feature representation *z*_0_ was derived from *s*_0_ based on relative positional encoding. After each layer of IPA‐Transformer transformation, the 1D vector representation was updated through a fully connected network and subsequently updated the pairwise features via cross product.

The objective of score‐matching networks differs from conventional neural network training goals. It does not aim to fit protein conformations directly but rather the scores of data perturbed to a certain degree, i.e., fitting the distribution of perturbed data. To measure how well the predicted scores fit the actual distribution, the DSM loss was computed as follows:

(7)

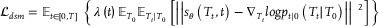




To ensure that the DSM loss at all time steps *t* results in a perfect fit score of 1, ensuring equal contribution of each time step to the loss function, the weights were set as follows:

(8) $$\lambda \;\left(t\right)=\frac{1}{E\left[\nabla_{T_{t}}logp_{t|0}\left(T_{t}|T_{0}\right)\right]}$$

Additionally, to ensure the model learns detailed features of protein structures, apart from the DSM loss on rotation and translation matrices, mean square error (MSE) supervision was also incorporated for the positions of backbone atoms and differences on the distance matrix for samples with fewer forward diffusion steps ($t<\frac{T}{4}$). Therefore, the complete network training loss function can be represented as:

(9) $$L=L_{dsm}+\omega_{1}L_{bb}+\omega_{2}L_{dist}$$

where ω_1_  = ω_2_  =  0.25 control the weight of conformation quality loss.

During the training process, with a maximum time step *T*  =  1.0, the DenoisingIPA was optimized using an Adam optimizer with the learning rate of 10^−4^.^[^ ^68^ ^]^ For the translation vector part of the network training, a linear noise strategy was employed within the VP‐SDE framework, while for the rotation matrix part, we use a logarithmic noise strategy within the VE‐SDE framework, as shown in the following equation^[^ ^66^ ^]^:

(10) $$\beta_{min}+\frac{t}{T}\left(\beta_{max}-\beta_{min}\right),\;\;\beta_{min}=0.1,\;\beta_{max}=20$$

(11) $$\sigma \;\left(t\right)=\log\left(te^{\sigma_{max}}+\left(T-t\right)e^{\sigma_{min}}\right)\;,\;\;\sigma_{min}=0.1,\;\sigma_{max}=1.5$$

### Formulation of IDPFold—Implementation Details

The training of IDPFold consists of two stages: pre‐training on experimental structures and fine‐tuning on MD trajectories. In **Table** **2**, the model hyperparameters utilized during the 2 training stages are presented.

**Table 2:** Hyperparameters and training details of IDPFold.

Hyperparameters	Training Stage	
	Training on Experimental Data	Training on MD Trajectories
Single Repr. Channel	256	
Pair Repr. Channel	128	
Hidden Channel	256	
IPA Layers	4	
Transformer Layers	2	
Transformer Heads	8	
Model Size	17.8 M parameters	
Learning Rate	10^−4^	10^−5^
Batch Size	8	32
Iterations	1.12 M	0.44 M
Time	≈9 GPU days	≈15 GPU days

### Evaluation Metrics and Analysis Tools

The quality of IDPFold‐predicted IDP conformational ensembles was evaluated from two perspectives: local features of the generated structures and global features of the conformational ensembles. For local features, biotite is used to compute inter‐residue bond lengths and bond angles of all generated conformations.^[^ ^69^ ^]^ The distribution of backbone dihedral angles was also analyzed to assess the model's prediction accuracy regarding *C*_α_ chirality and secondary structure. Additionally, mdtraj was used to calculate scalar coupling between HN and *H*_α_, employed SPARTA+ to compute chemical shifts, and then performed ensemble averaging.^[^ ^70^, ^71^ ^]^ These analyses helped determine whether the local environment of the protein backbone aligns with experimental observations.

For global features, the Rg of the generated conformations was calculated, and the ensemble average was compared with experimental results. Additionally, the RMSD of generated conformations against the initial structures used in MD simulation was calculated to construct Rg‐RMSD space. Through clustering the generated conformations and projecting them onto Rg‐RMSD space, the diversity of generated conformations is analyzed, and the model's learning efficacy in capturing the Boltzmann distribution information from MD trajectories in the training dataset is assessed. MMTSB toolset is applied for confirmation clustering.

Due to the fact that most current work on generating protein conformational ensembles from sequences adopts a coarse‐grained representation, where only *C*_α_ coordinates are generated, the generated structures were first fixed with pdbfixer and ran a 100‐step energy minimization. During minimization, restraints were added on *C*_α_ to make sure the optimized backbones did not differ from the original ones too much. This process cost ≈6 s per conformation. Validity and Fidelity were then evaluated, and the performance of IDPFold was compared with previous methods.

Validity assesses whether the generated conformations contain unreasonable *C*_α_ distances. In a protein structure, due to van der Waals interactions between atoms, *C*_α_ distances should not be too close. Additionally, because neighboring *C*_α_ in the protein backbone were connected by *C* − *N* with specific bond lengths, *C*_α_ distances should not be excessively far apart. Therefore, following the approach of Str2Str, a reasonable range for *C*_α_ distances was defined as follows:

(12) $$\delta_{vdw}<d_{C_{\alpha }^{i},C_{\alpha }^{i+1}}<\delta_{bond}$$

where δ_*vdw*_ =  2 × 1.7 − 0.4 was defined as the sum of two *C*_α_ van der Waals radius minus an acceptable overlap distance of 0.4.^[^ ^72^ ^]^ δ_*bond*_ was taken as the maximum *C*_α_ distance observed in MD trajectory of each test system. Since a reasonable range was defined, validity was defined as proportion of valid conformations. A higher validity indicates a lower probability of mis‐estimated *C*_α_ distance, thus demonstrating better performance.

Fidelity refers to how well the model‐generated ensembles match experimental observations. Rg, *C*_α_ and *C*_β_ secondary chemical shifts, J‐coupling constants between *HN* and *H*_α_, and backbone N‐HN RDCs were selected as target physical quantities to measure the fidelity of models, calculating the errors between model‐generated conformational ensembles and experimental observations using the following formula:

(13) $$\varepsilon_{Rg}=\frac{{\langle Rg\rangle }^{pred}-{\langle Rg\rangle }^{exp}}{{\langle Rg\rangle }^{exp}}$$

(14) $$RMS\;D_{\langle A\rangle }=\sqrt{E{\langle A\rangle }^{pred}-{\left({\langle A\rangle }^{exp}\right)}^{2}}$$

where 〈*Rg*〉 denotes the ensemble average Rg, 〈*Rg*〉^*pred*^ was the generated ensemble average Rg while 〈*Rg*〉^*exp*^ was an experimental observation. Similarly, 〈*A*〉^*pred*^ and 〈*A*〉^*exp*^ denotes ensemble average physical quantity (e.g., chemical shifts and J‐couplings) and experimental observation, respectively. Using the above definitions, we calculated ε_*Rg*_, $RMSD_{\delta C_{\alpha }}$, $RMSD_{\delta C_{\beta }}$, 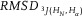 and $RMSD_{RDC_{NH}}$ for benchmarking current conformation generation methods, aiming for values close to zero for all. ε_*Rg*_ reflects the reasonableness of the model's generated conformations in terms of their compactness, while *RMSD*s on other physical quantities indicates how well the generated conformations conform to experimental observations on local structures.

For evaluating the conformational ensembles generated by the models, all methods except AF‐cluster generated 300 conformations for subsequent evaluation.^[^ ^73^, ^74^ ^]^ This number of conformations has been previously shown to adequately reflect the structural diversity for intrinsically disordered proteins of similar size to the largest protein in our test set. Additionally, a convergence test was conducted on IDPFold‐generated ensembles as depicted in Figure S18 (Supporting Information). The number of conformations generated by AF‐cluster is influenced by the number of clusters in the MSA, and the default MSA and clustering settings were used. All conformation generation was performed on NVIDIA A100 GPUs.

### Statistical Analysis

Data are presented as means ± SDs or distributions. The sample size (n) for each analysis was fixed and specified in the preceding paragraph. All statistical analyses were performed using Python 3.9 with the SciPy and NumPy libraries. MD trajectories and generated conformations are loaded and analyzed with MDTraj and Biotite. A two‐sided paired *t*‐test was used to compare the means of two related groups. Statistical significance was determined by specific p‐values, with *p*<0.05 considered significant, and these values were reported in the main text and Figure legends.

## Conflict of Interest

The authors declare no conflict of interest.

## Supporting information



Supporting Information

Supplemental Movie 1

## Data Availability

Raw data for training and evaluating IDPFold is publicly available from PDB (http://www.rcsb.org/) and IDRome (KULL‐Centre/_2023_Tesei_IDRome) databases. Processed data will be shared upon request.
