# Supplementary material for: Accurate Generation of Conformational Ensembles for Intrinsically Disordered Proteins with IDPFold
Source: Adv Sci (Weinh). 2025 Oct 13;12(48):e11636. doi: 10.1002/advs.202511636 (PMC12752595; doi:10.1002/advs.202511636)
Supplement: Supplementary file 1 — Supporting Information [file ADVS-12-e11636-s001.docx]

Supporting Information

**Accurate Generation of Conformational Ensembles for Intrinsically Disordered Proteins with IDPFold**

Junjie Zhu^1,#^, Zhengxin Li^1,#^, Zhuoqi Zheng^1^, Bo Zhang^1^, Bozitao Zhong^1^, Jie Bai^1^, Xiaokun Hong^2^, Taifeng Wang^3^, Ting Wei^1,*^, Jianyi Yang^4,*^, Hai-Feng Chen^1,*^

^1^State Key Laboratory of Microbial Metabolism, Department of Bioinformatics and Biostatistics, SJTU-Yale Joint Center for Biostatistics, National Experimental Teaching Center for Life Sciences and Biotechnology, School of Life Sciences and Biotechnology, Shanghai Jiao Tong University, 200240, Shanghai, China.

^2^College of Biological Science and Engineering, Fuzhou University, Fuzhou 350116, Fujian, China

^3^BioMap,10 Beilun Industrial Park, Yongteng North Road, 100080, Beijing, China.

^4^MOE Frontiers Science Center for Nonlinear Expectations, Research Center for Mathematics and Interdisciplinary Sciences, Shandong University, 266237, Qingdao, China.

***Corresponding Author**

**Email**: haifengchen@sjtu.edu.cn; [yangjy@sdu.edu.cn](mailto:yangjy@sdu.edu.cn); weitinging@sjtu.edu.cn

**Notes**

The authors declare that there is no conflict of interest.

**Author Contributions**

^#^These authors contributed equally to this work.

**Supporting video caption**

**Movie S1**. Alignment of dynamic structure of IDPFold generation for ubiquitin.

**Table S1.** IDP systems in test set, $R_{g}^{exp}$, experimental chemical shifts, J-couplings and RDCs. Abbreviated forms of long system names are marked in bold.

| **System** | $\boldsymbol{R}_{\boldsymbol{g}}^{\boldsymbol{exp}}$ | **Chemical Shifts** | **J-couplings and RDCs** |
| --- | --- | --- | --- |
| Sic1 | 34.7^1^ | $C_{\alpha}$, $C_{\beta}$ chemical shifts^1^ | N/A |
| Aβ 42 | 12.4^2^ | $C_{\alpha}$, $C_{\beta}$ chemical shifts^3^ | ${}^{3}{J(H_{N},H_{\alpha})}$ scalar coupling^4^, backbone N-HN RDCs^5^ |
| Aβ 40 | 12^6^ | $C_{\alpha}$, $C_{\beta}$ chemical shifts^3^ | ${}^{3}{J(H_{N},H_{\alpha})}$ scalar coupling^4^, backbone N-HN RDCs^5^ |
| RS1 | 12.62^7^ | $C_{\alpha}$ chemical shifts^8^ | ${}^{3}{J(H_{N},H_{\alpha})}$ scalar coupling^8^, backbone N-HN RDCs^8^ |
| Histatin5 | 13.8^9^ | N/A | ${}^{3}{J(H_{N},H_{\alpha})}$ scalar coupling^10^ |
| p15^PAF^ | 28.1^11^ | $C_{\alpha}$, $C_{\beta}$ chemical shifts^11^ | backbone N-HN RDCs^11^ |
| FEZ1 monomer | 36^12^ | N/A | N/A |
| HIV-1 Tat_133_ | 33^13^ | N/A | N/A |
| R17 | 22.9^14^ | N/A | N/A |
| PIR domain | 26.5^15^ | N/A | N/A |
| IB5 | 27.9^16^ | N/A | N/A |
| PaaA2 | 22.4^17^ | $C_{\alpha}$, $C_{\beta}$ chemical shifts^17^ | backbone N-HN RDCs^17^ |
| ACTR | 25^18^ | $C_{\alpha}$, $C_{\beta}$ chemical shifts^19^ | backbone N-HN RDCs^20^ |
| drkN SH3 | 16.7^21^ | $C_{\alpha}$, $C_{\beta}$ chemical shifts^22^ | ${}^{3}{J(H_{N},H_{\alpha})}$ scalar coupling^7^ |
| Juxtanodin | 55.9^23^ | N/A | N/A |
| α Synuclein | 33^24^ | $C_{\alpha}$, $C_{\beta}$ chemical shifts^19^ | ${}^{3}{J(H_{N},H_{\alpha})}$ scalar coupling^25^ |
| β Synuclein | 49^26^ | N/A | N/A |
| γ Synuclein | 61^26^ | N/A | N/A |
| Tau K18 | 38^27^ | N/A | N/A |
| Tau K19 | 35^27^ | N/A | N/A |
| Prothymosin α | 37.8^28^ | N/A | N/A |
| p53 (1-93) **[p53]** | 28.7^29^ | $C_{\alpha}$, $C_{\beta}$ chemical shifts^29^ | N/A |
| Human NCBD domain **[NCBD]** | 33^30^ | N/A | N/A |
| Human Calpastatin (137-237) **[Calpastatin]** | 39^30^ | N/A | N/A |
| N-term NRG1 type III **[NtermNRG1]** | 26.8^31^ | N/A | N/A |
| N-term VS Virus phosphoprotein  **[NtermVS]** | 26^32^ | N/A | N/A |
| E3 ubiquitin ligase RNF4 (32-82)  **[RNF4]** | 25.8^33^ | N/A | N/A |

**Table S2.** Sequences of IDPs in test set.

| **System** | **Sequence** |
| --- | --- |
| Sic1 | MTPSTPPRSRGTRYLAQPSGNTSSSALMQGQKTPQKPSQNLVPVTPSTTKSFKNAPLLAPPNSNMGMTSPFNGLTSPQRSPFPKSSVKRTLF |
| Aβ 42 | DAEFRHDSGYEVHHQKLVFFAEDVGSNKGAIIGLMVGGVVIA |
| Aβ 40 | DAEFRHDSGYEVHHQKLVFFAEDVGSNKGAIIGLMVGGVV |
| RS1 | GAMGPSYGRSRSRSRSRSRSRSRS |
| Histatin5 | DSHAKRHHGYKRKFHEKHHSHRGY |
| p15^PAF^ | VRTKADSVPGTYRKVVAARAPRKVLGSSTSATNSTSVSSRKAENKYAGGNPVCVRPTPKWQKGIGEFFRLSPKDSEKENQIPEEAGSSGLGKAKRKACPLQPDHTNDEKE |
| FEZ1 monomer | QIQEEEETLQDEEVWDALTDNYIPSLSEDWRDPNIEALNGNCSDTEIHEKEEEEFNEKSENDSGINEEPLLTADQVIEEIEEMMQNSPDPEEEEEVLEEEDGG |
| HIV-1 Tat_133_ | MEPVDPRLEPWKHPGSQPRTACTNCYCKKCCFHCQVCFIRKALGISYGRKKRRQRRRAPPDSETHQVSPPKQPASQPRGDPTGPKESKKKVERETETHPVN |
| R17 | RLEESLEYQQFVANVEEEEAWINEKMTLVASEDYGDTLAAIQGLLKKHEAFETDFTVHKDRVNDVAANGEDLIKKNNHHVENITAKMKGLKGKVSDLEKA |
| PIR domain | SVSPMRSVSENSLVAMDFSGQKTRVIDNPTEALSVAVEEGLAWRKKGCLRLGNHGSPTAPSQSSAVNMALHRSQP |
| IB5 | SARSPPGKPQGPPQQEGNKPQGPPPPGKPQGPPPAGGNPQQPQAPPAGKPQGPPPPPQGGRPPRPAQGQQPPQ |
| PaaA2 | MDYKDDDDKNRALSPMVSEFETIEQENSYNEWLRAKVATSLADPRPAIPHDEVERRMAERFAKMRKERSKQ |
| ACTR | GTQNRPLLRNSLDDLVGPPSNLEGQSDERALLDQLHTLLSNTDATGLEEIDRALGIPELVNQGQALEPKQD |
| drkN SH3 | MEAIAKHDFSATADDELSFRKTQILKILNMEDDSNWYRAELDGKEGLIPSNYIEMKNHD |
| Juxtanodin | MTDTPVTLSGSECNGDRPPENGQQPSSQTRKTTDADETQTYYGVEPSLQHLPAKENQEESGNSKGNVLPRGSEDEKILNENTEENLFVVHQAIQDLSLQETSAEDTVFQEGHPWKKIPLNSHNLDMSRQKERIVHQHLEQREDESAAHQATEIEWLGFQKSSQVDILHSKCDEEEEVWNEEINEEDVDECAEDEGEDEVRVIEFKRKYREGSPLKEESLAREDSPLSSPSSQPGTPDEQLVLGKKGDIARNSYSRYNTISYRKIRKGNTKQRIDEFESMMHL |
| α Synuclein | MDVFMKGLSKAKEGVVAAAEKTKQGVAEAAGKTKEGVLYVGSKTKEGVVHGVATVAEKTKEQVTNVGGAVVTGVTAVAQKTVEGAGSIAAATGFVKKDQLGKNEEGAPQEGILEDMPVDPDNEAYEMPSEEGYQDYEPEA |
| β Synuclein | MDVFMKGLSMAKEGVVAAAEKTKQGVTEAAEKTKEGVLYVGSKTREGVVQGVASVAEKTKEQASHLGGAVFSGAGNIAAATGLVKREEFPTDLKPEEVAQEAAEEPLIEPLMEPEGESYEDPPQEEYQEYEPEA |
| γ Synuclein | MDVFKKGFSIAKEGVVGAVEKTKQGVTEAAEKTKEGVMYVGAKTKENVVQSVTSVAEKTKEQANAVSEAVVSSVNTVATKTVEEAENIAVTSGVVRKEDLRPSAPQQEGEASKEKEEVAEEAQSGGD |
| Tau K18 | QTAPVPMPDLKNVKSKIGSTENLKHQPGGGKVQIINKKLDLSNVQSKCGSKDNIKHVPGGGSVQIVYKPVDLSKVTSKCGSLGNIHHKPGGGQVEVKSEKLDFKDRVQSKIGSLDNITHVPGGGNKKIE |
| Tau K19 | QTAPVPMPDLKNVKSKIGSTENLKHQPGGGKVQIVYKPVDLSKVTSKCGSLGNIHHKPGGGQVEVKSEKLDFKDRVQSKIGSLDNITHVPGGGNKKIE |
| Prothymosin α | MSDAAVDTSSEITTKDLKEKKEVVEEAENGRDAPANGNAENEENGEQEADNEVDEEEEEGGEEEEEEEEGDGEEEDGDEDEEAESATGKRAAEDDEDDDVDTKKQKTDEDD |
| p53 (1-93) | MEEPQSDPSVEPPLSQETFSDLWKLLPENNVLSPLPSQAMDDLMLSPDDIEQWFTEDPGPDEAPRMPEAAPPVAPAPAAPTPAAPAPAPSWPL |
| Human NCBD domain | ISPLKPGTVSQQALQNLLRTLRSPSSPLQQQQVLSILHANPQLLAAFIKQRAAKYANSNPQPIPGQPGMPQGQPGLQPPTMPGQQGVHSNPAMQNMNPMQAGVQR |
| Human Calpastatin (137-237) | AVPVESKPDKPSGKSGMDAALDDLIDTLGGPEETEEENTTYTGPEVSDPMSSTYIEELGKREVTIPPKYRELLAKKEGITGPPADSSKPIGPDDAIDALSSDFTCGSPTAAGKKTEKEESTEVLKAQSAGTVRSAAPPQEK |
| N-term NRG1 type III | MEIYSPDMSEVAAERSSSPSTQLSADPSLDGLPAAEDMPEPQTEDGRTPGLVGLAV |
| N-term VS Virus phosphoprotein | MDNLTKVREYLKSYSRLDQAVGEIDEIEAQRAEKSNYELFQEDGVEEHTKPSYFQAADDSHHHHHHHH |
| E3 ubiquitin ligase RNF4 (32-82) | EAEPIELVETAGDEIVDLTCESLEPVVVDLTHNDSVVIVDERRRPRRNARR |

**Table S3.** Performance of all 8 versions of AlphaFlow.

| **Methods** | **Validity (↑)** | **Fidelity** | |
| --- | --- | --- | --- |
|  |  | $\varepsilon_{Rg}$ | ${MAE}_{\delta C_{\alpha}}$ (↓) |
| AF-PDB-base | **0.97** | $\boldsymbol{-0.24}$ | **0.54** |
| AF-PDB-distilled | 0.91 | $-0.33$ | 0.78 |
| AF-MD-base | 0.94 | $-0.32$ | **0.54** |
| AF-MD-distilled | 0.91 | $-0.44$ | 0.66 |
| ESM-PDB-base | 0.94 | $\boldsymbol{-0.24}$ | 0.64 |
| ESM-PDB-distilled | 0.89 | $-0.33$ | 0.77 |
| ESM-MD-base | 0.96 | $-0.35$ | 0.62 |
| ESM-MD-distilled | 0.90 | $-0.47$ | 0.68 |


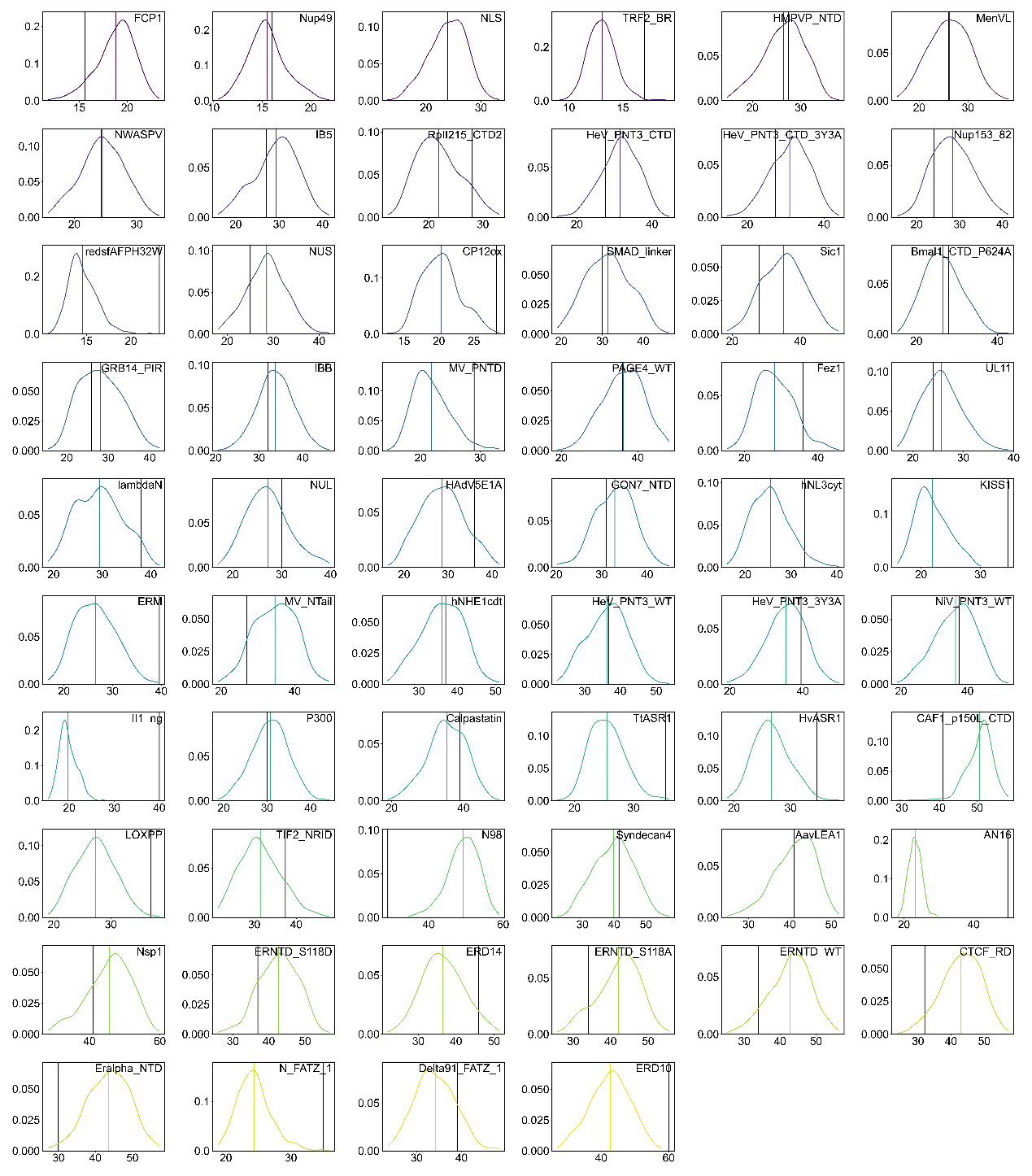


**Figure S1.** Rg distribution of IDPFold generated conformation ensembles (colored) on 58 IDP systems from IDRome. Experimental Rg values are plotted as black solid lines.

**
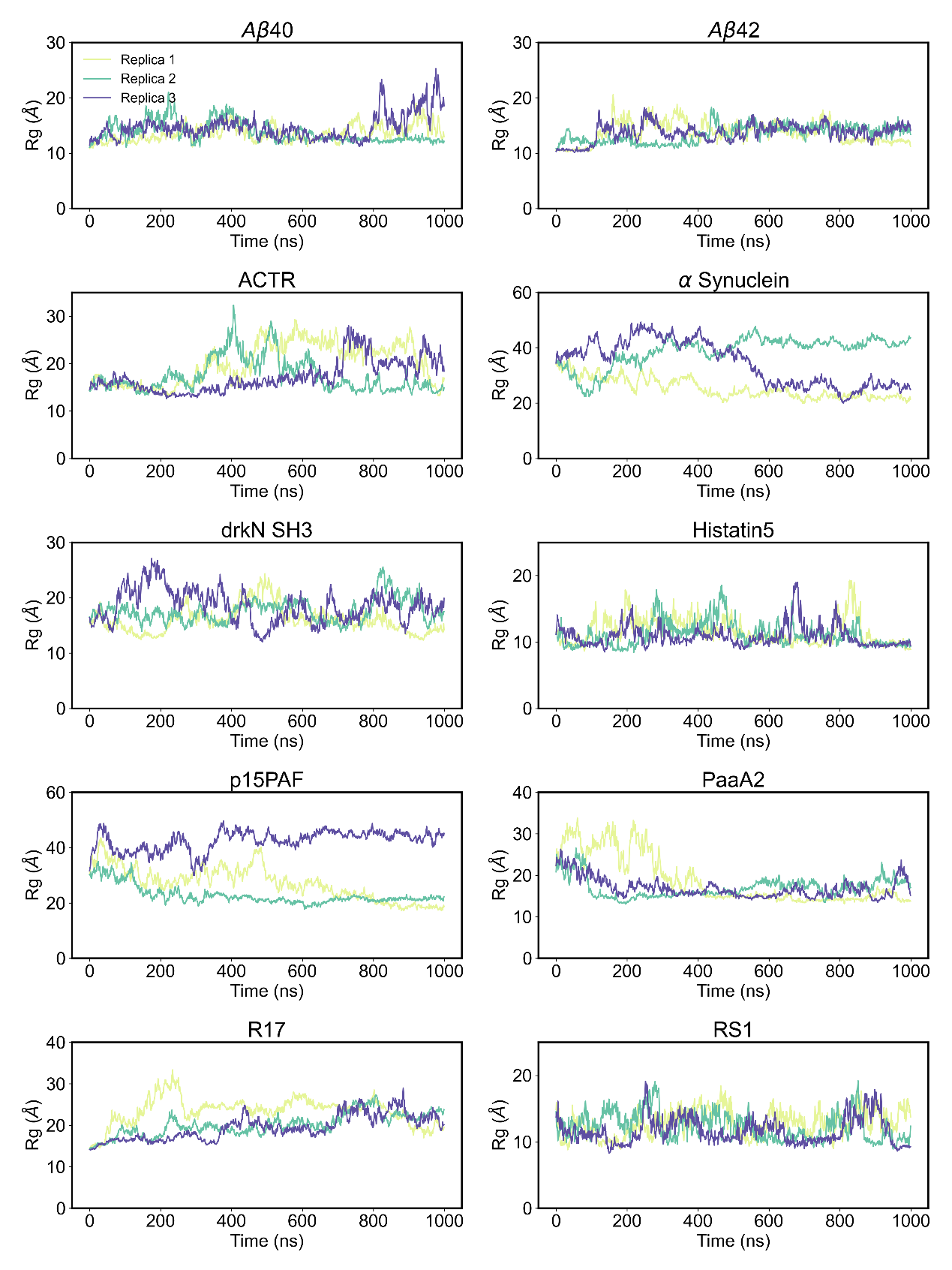
**

**Figure S2.** Convergence of all-atom MD simulation.


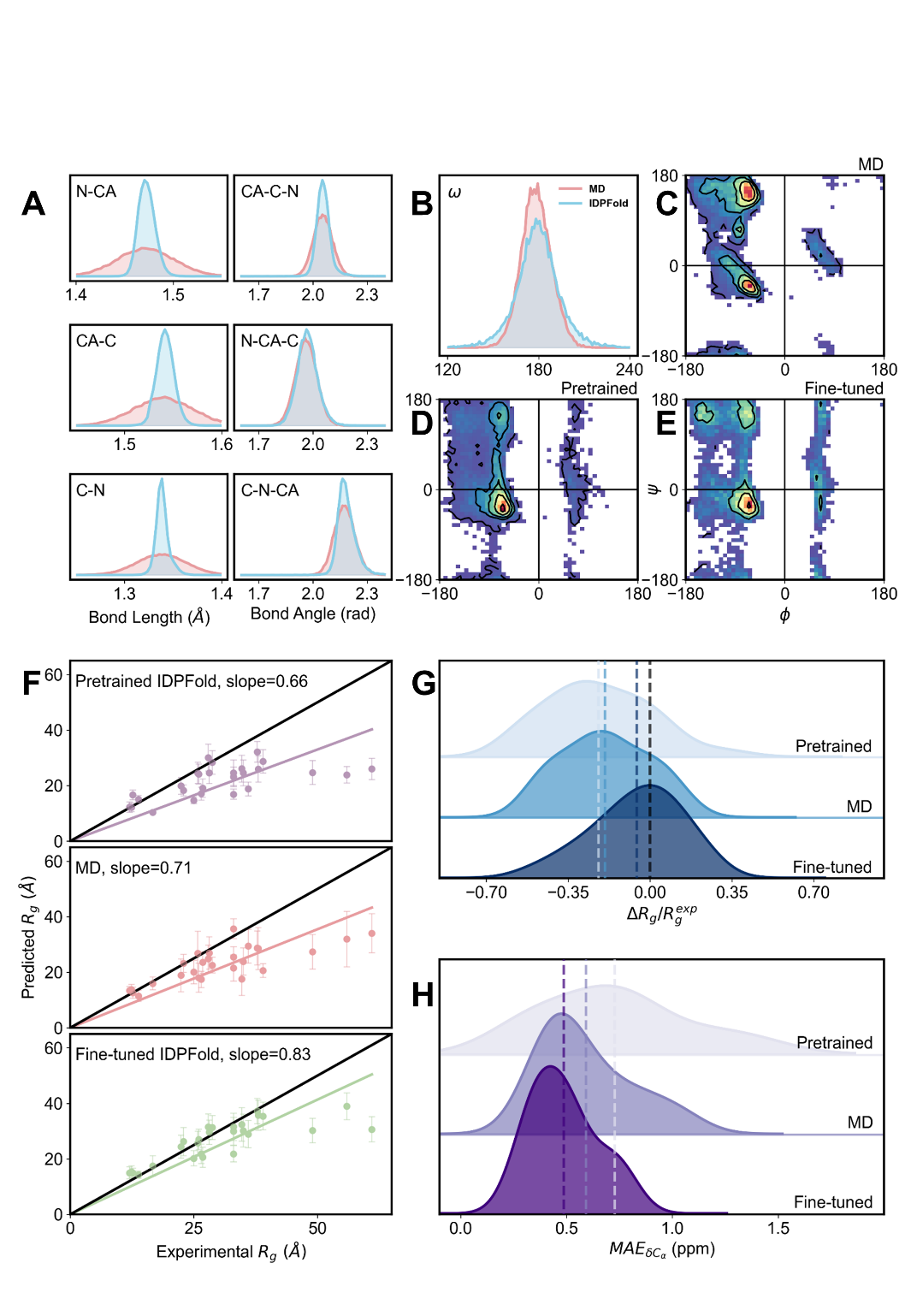


**Figure S3:** Comparison between the generated ensembles of IDPFold and all-atom MD simulation with ESFF1. **A, B,** Bond length, bond angle **(A)** and omega angle **(B)** distributions of IDPFold generated ensembles (colored) and MD simulation (black). **C-E,** Ramachandran plots of MD simulations **(C)**, pretrained IDPFold generated ensembles **(D)** and finetuned IDPFold generated ensembles **(E)**. **F,** Regression plot between experimental Rg values and predicted Rg values for MD simulation and IDPFold. The intercept was set to zero. **G, H,** Ensemble average Rg error **(G)**, RMSD on ensemble average $C_{\alpha}$ chemical shift **(H)** of MD trajectories (middle), pretrained (upper) and fine-tuned (lower) IDPFold against experimental values.


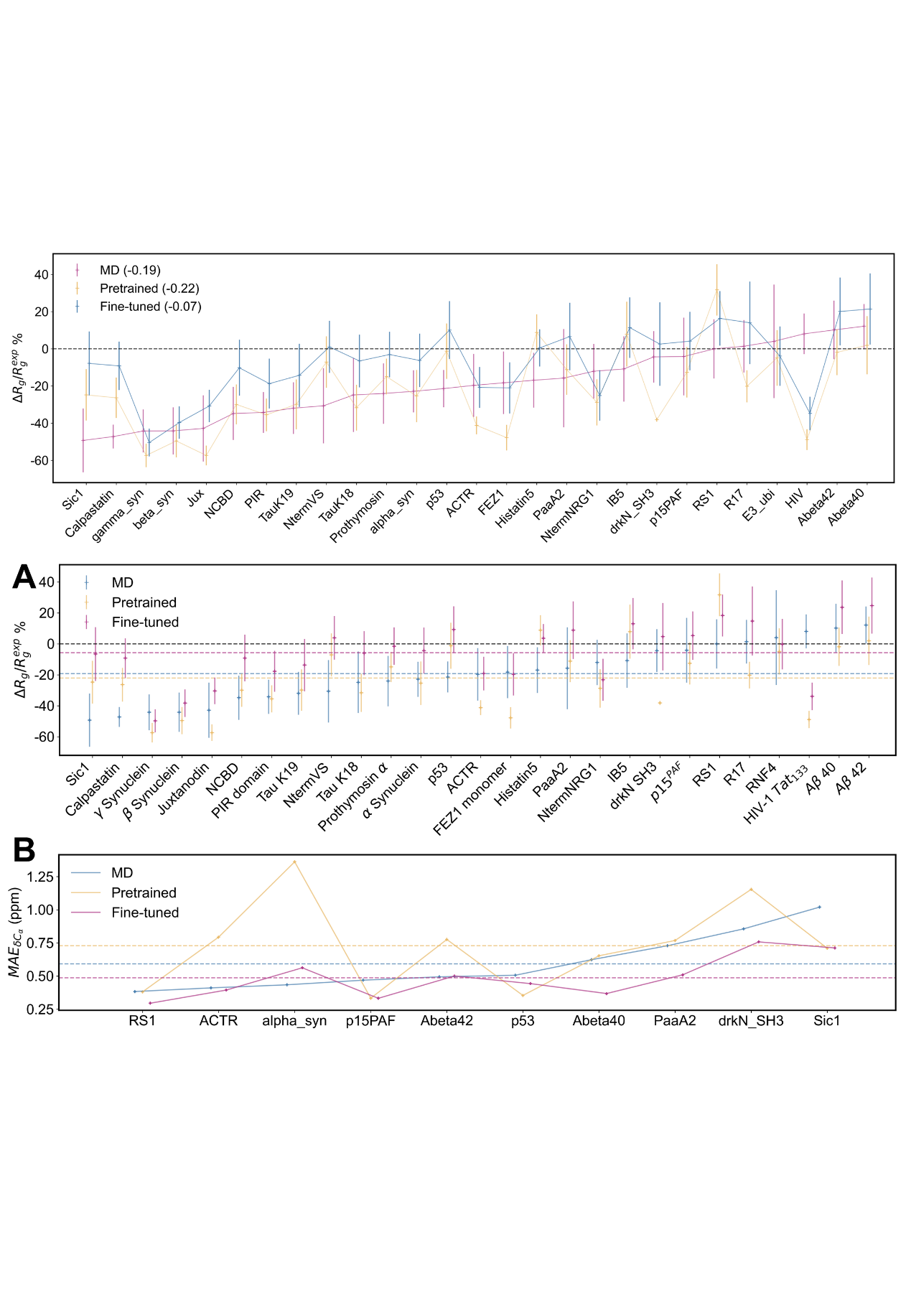


**Figure S4.** Differences between generated conformational ensembles and experimental observations. (A) Average Rg errors and their standard deviation on all 27 IDPs. (B) Mean average error on ensemble average $C_{\alpha}$ chemical shift on all 10 IDPs.


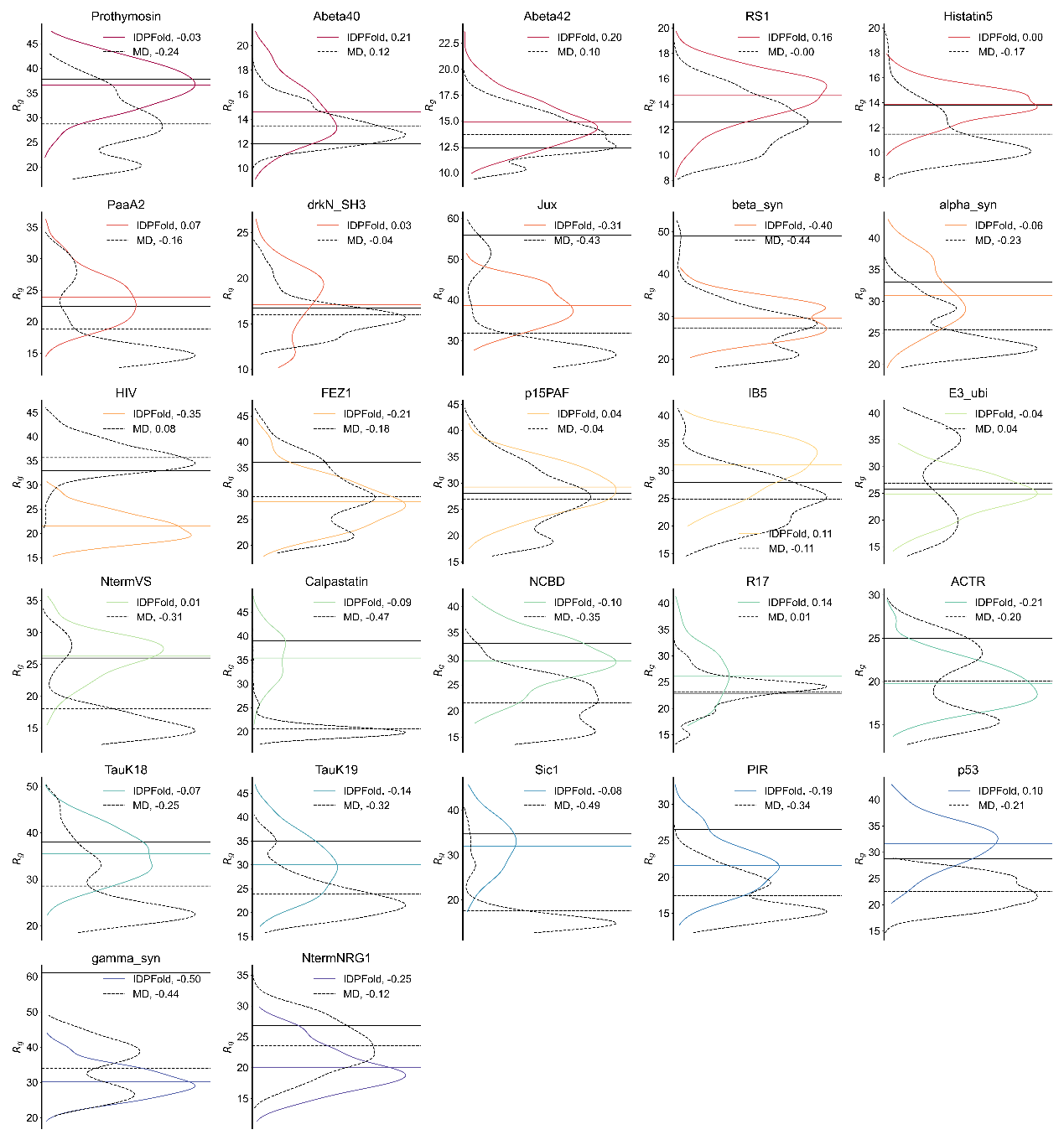


**Figure S5.** Rg distribution of **IDPFold** generated conformation ensembles (colored) and MD trajectories (black dashed) on 27 IDP systems. Experimental Rg values are plotted as black solid lines.


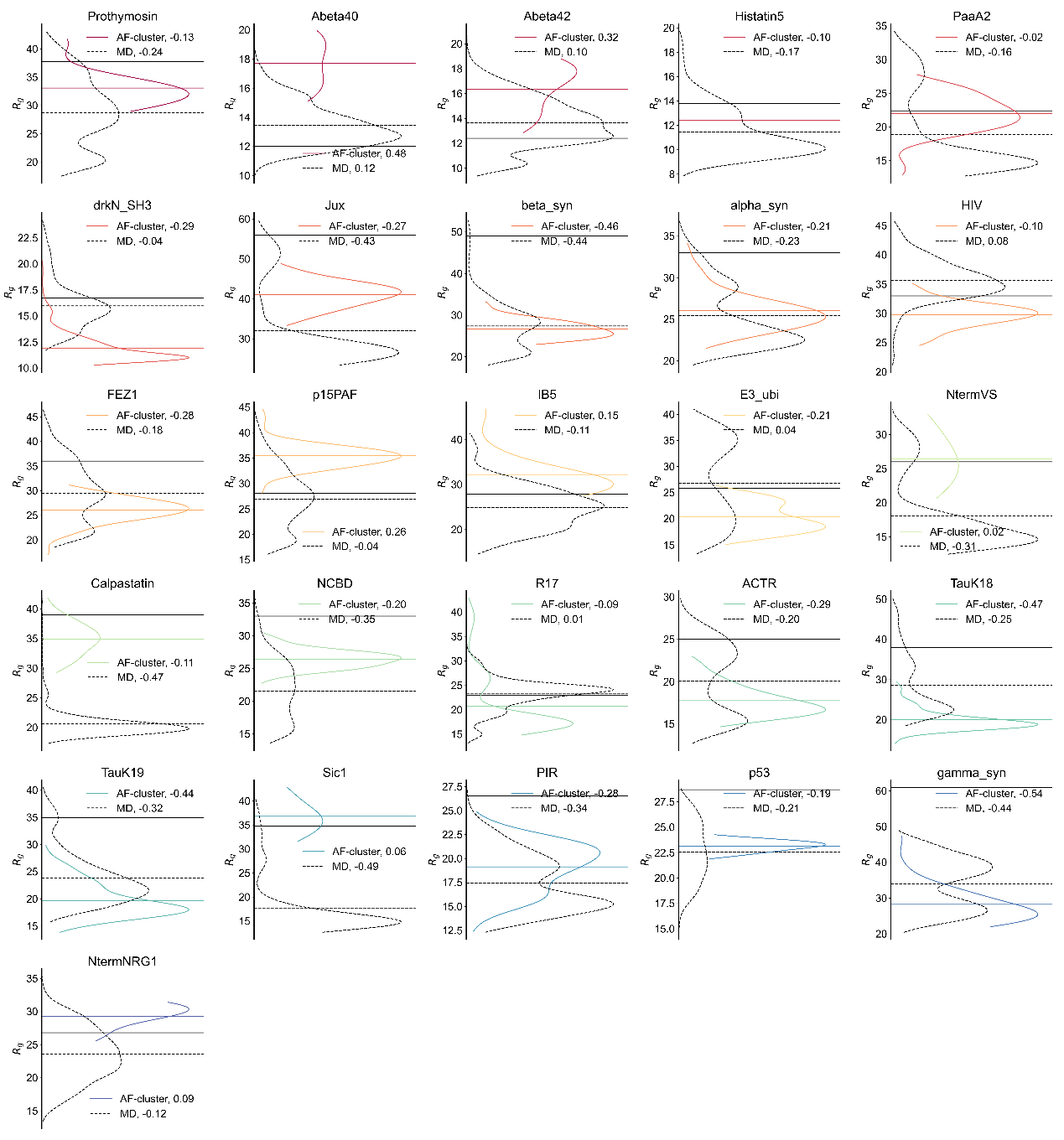


**Figure S6.** Rg distribution of **AF-cluster** generated conformation ensembles (colored) on 26 IDP systems. Experimental Rg values are plotted as black solid lines.


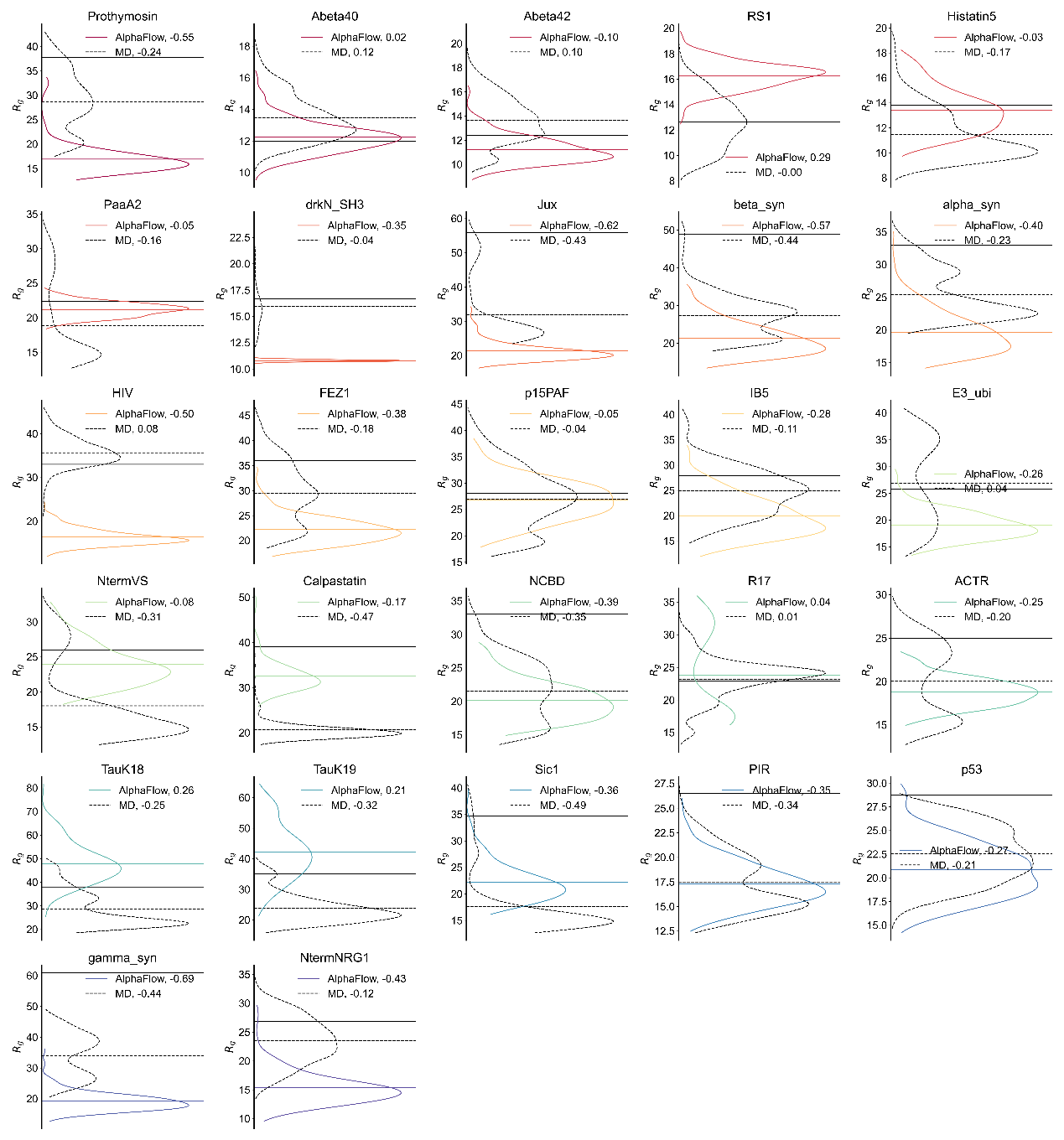


**Figure S7.** Rg distribution of **AlphaFlow** (PDB-base) generated conformation ensembles (colored) on 27 IDP systems. Experimental Rg values are plotted as black solid lines.


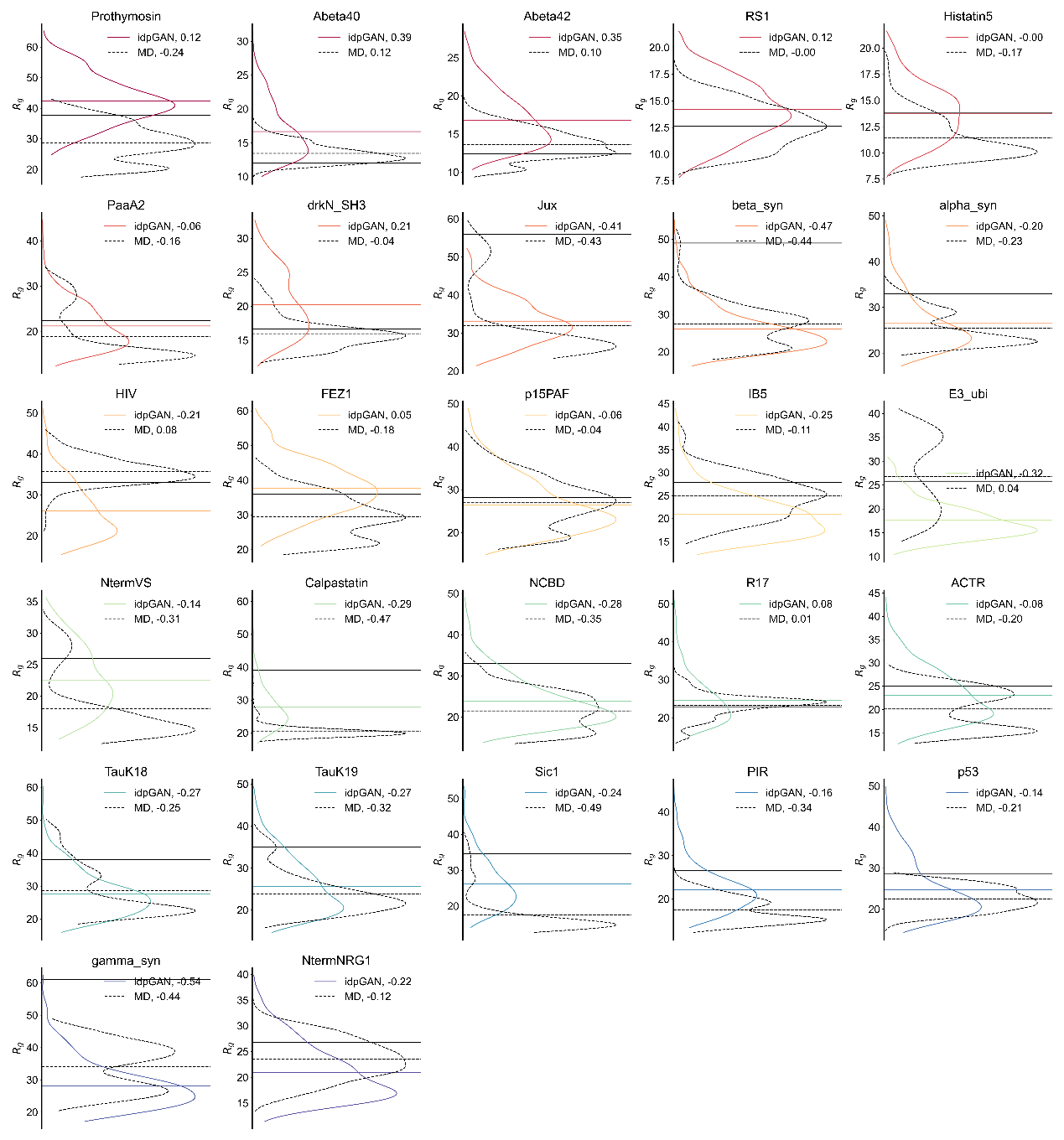


**Figure S8.** Rg distribution of **idpGAN** generated conformation ensembles (colored) on 27 IDP systems. Experimental Rg values are plotted as black solid lines.


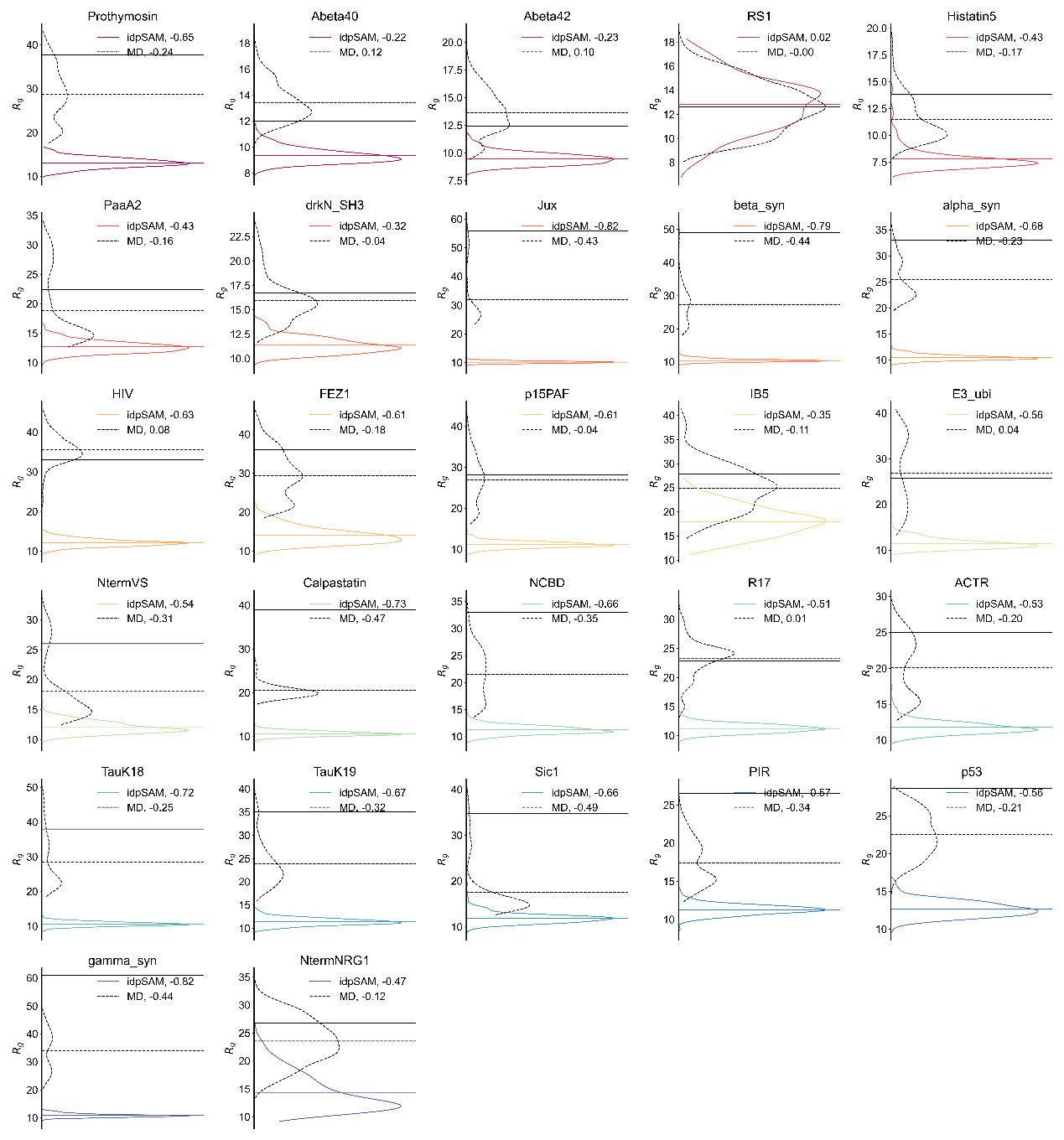


**Figure S9.** Rg distribution of **idpSAM** generated conformation ensembles (colored) on 27 IDP systems. Experimental Rg values are plotted as black solid lines.


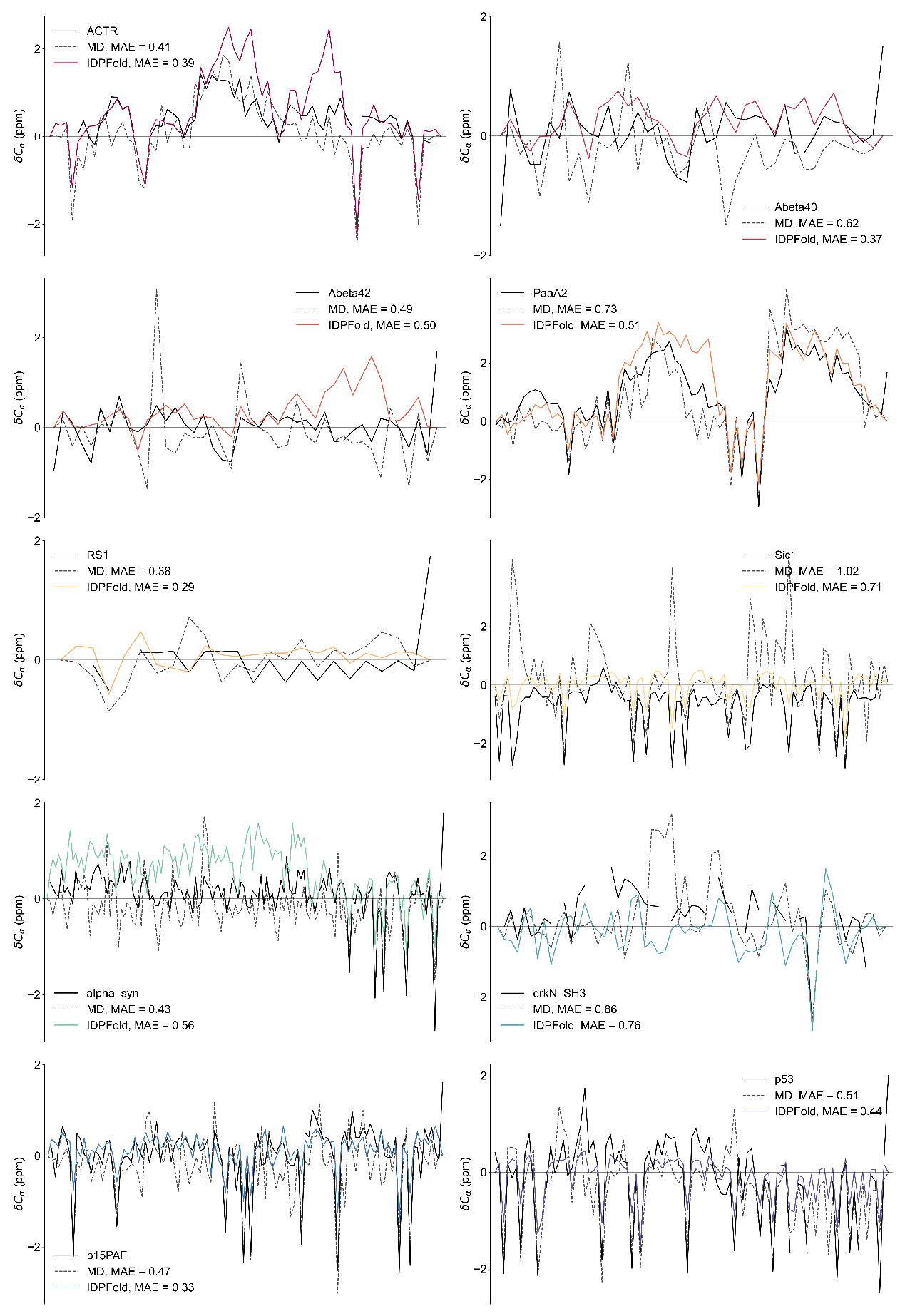


**Figure S10.** Ensemble average $C_{\alpha}$ chemical shifts of **IDPFold** generated conformation ensembles (colored) and MD trajectories (black dashed) on 10 IDP systems. Experimental values are plotted as black solid lines.


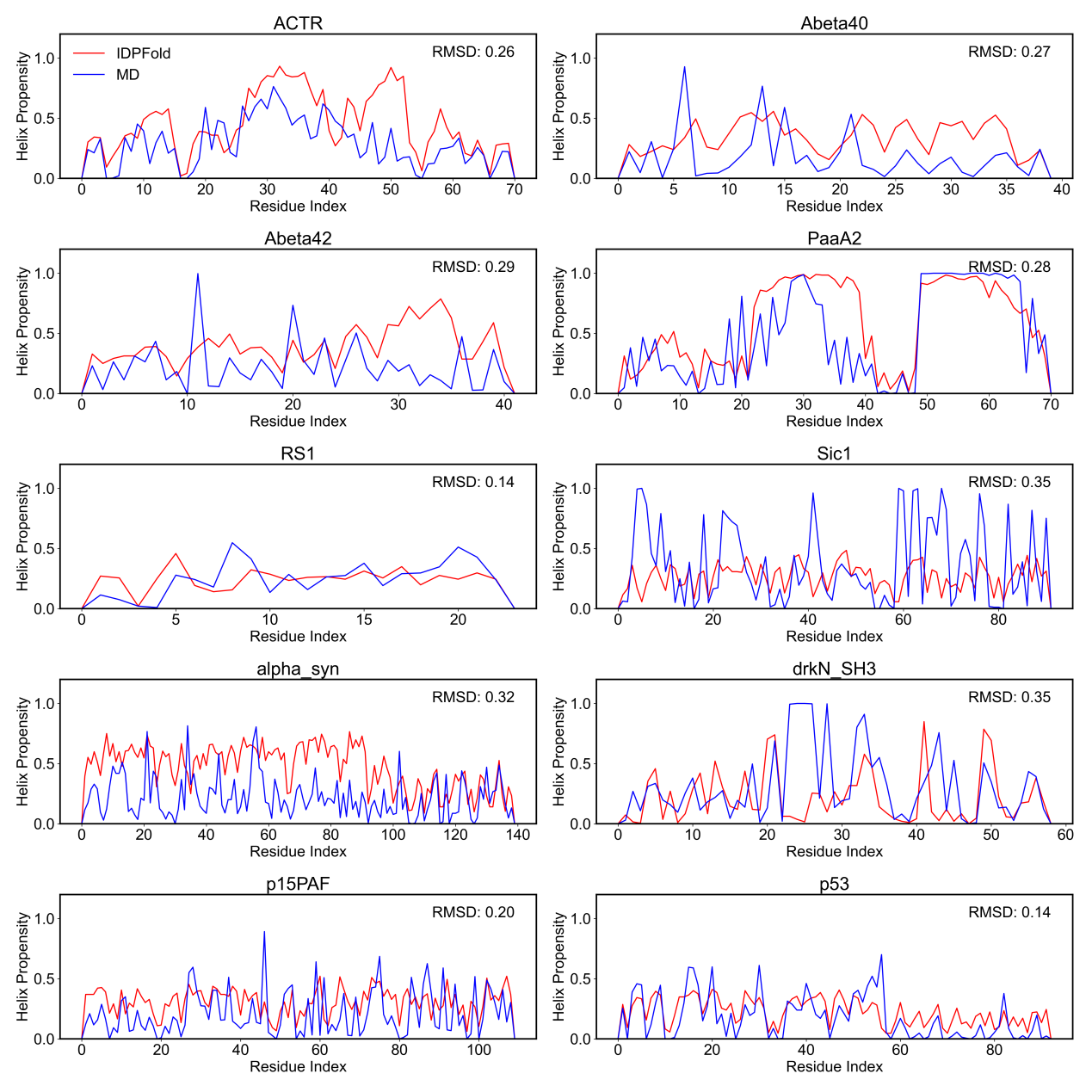


**Figure S11.** Helix propensity calculated from $C_{\alpha}$ chemical shifts for MD simulation and IDPFold.


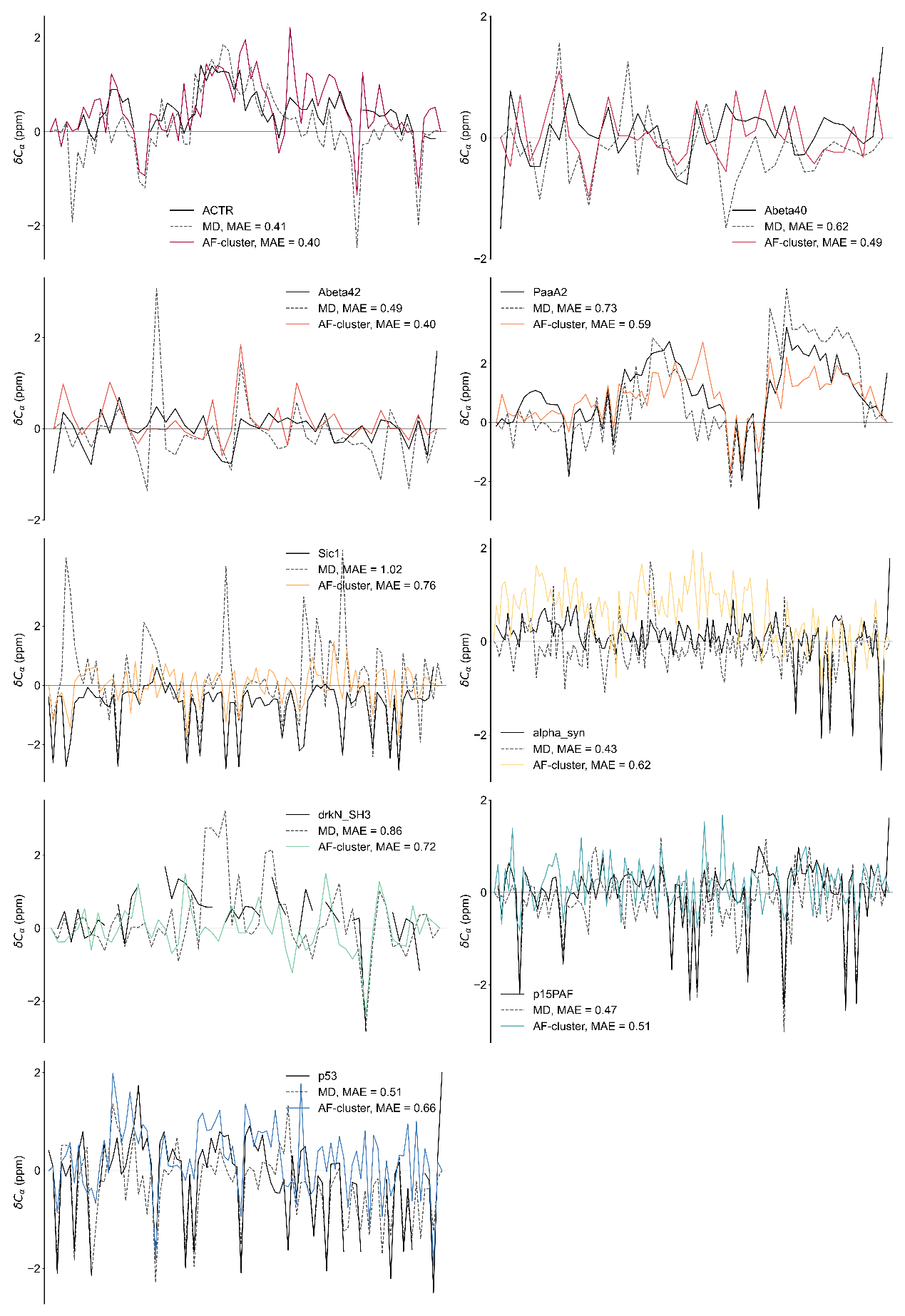


**Figure S12.** Ensemble average $C_{\alpha}$ chemical shifts of **AF-cluster** generated conformation ensembles (colored) on 10 IDP systems. Experimental values are plotted as black solid lines.


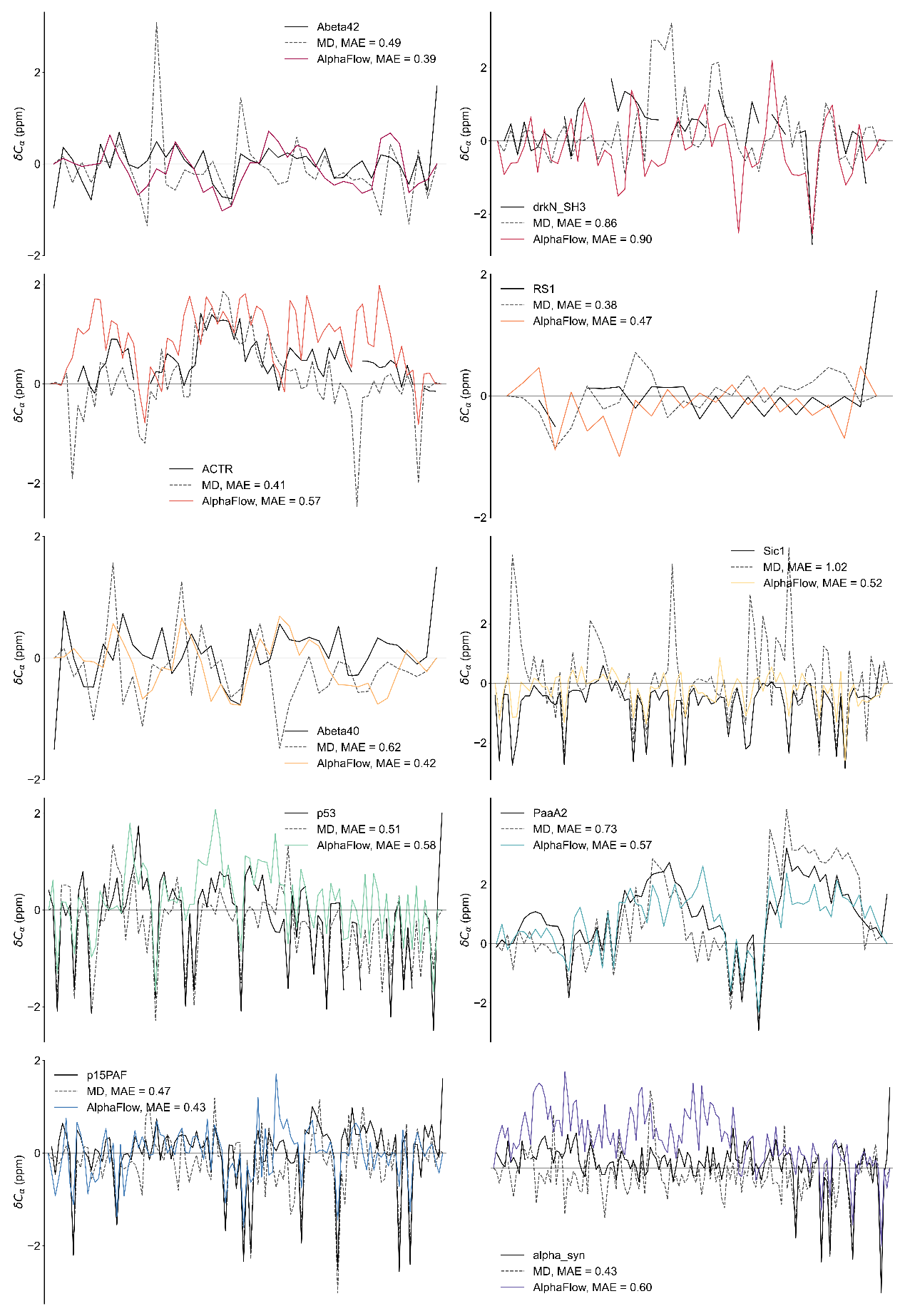


**Figure S13.** Ensemble average $C_{\alpha}$ chemical shifts of **AlphaFlow** (PDB-base) generated conformation ensembles (colored) on 10 IDP systems. Experimental values are plotted as black solid lines.


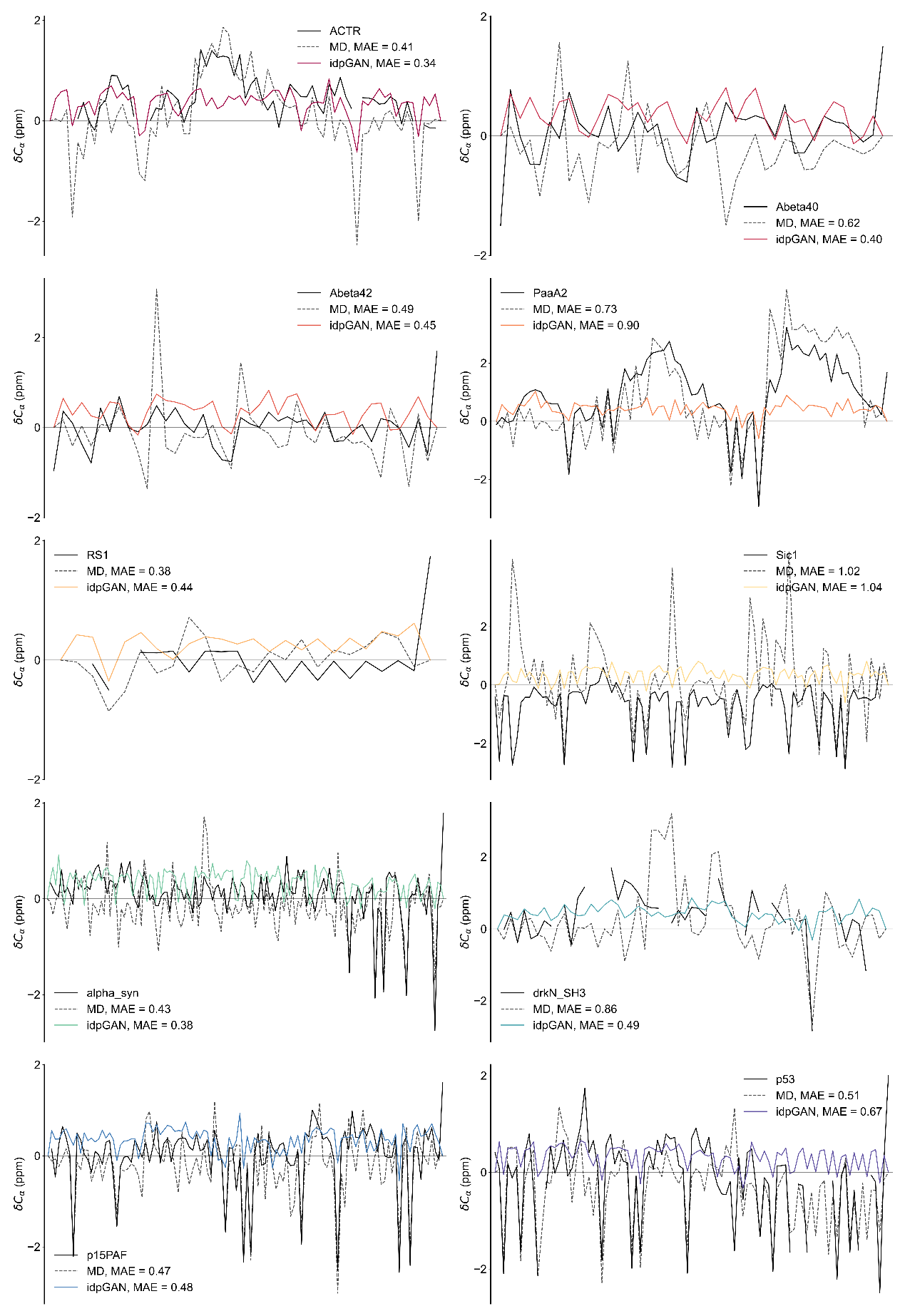


**Figure S14.** Ensemble average $C_{\alpha}$ chemical shifts of force-field-refined **idpGAN** generated conformation ensembles (colored) on 10 IDP systems. Experimental values are plotted as black solid lines.


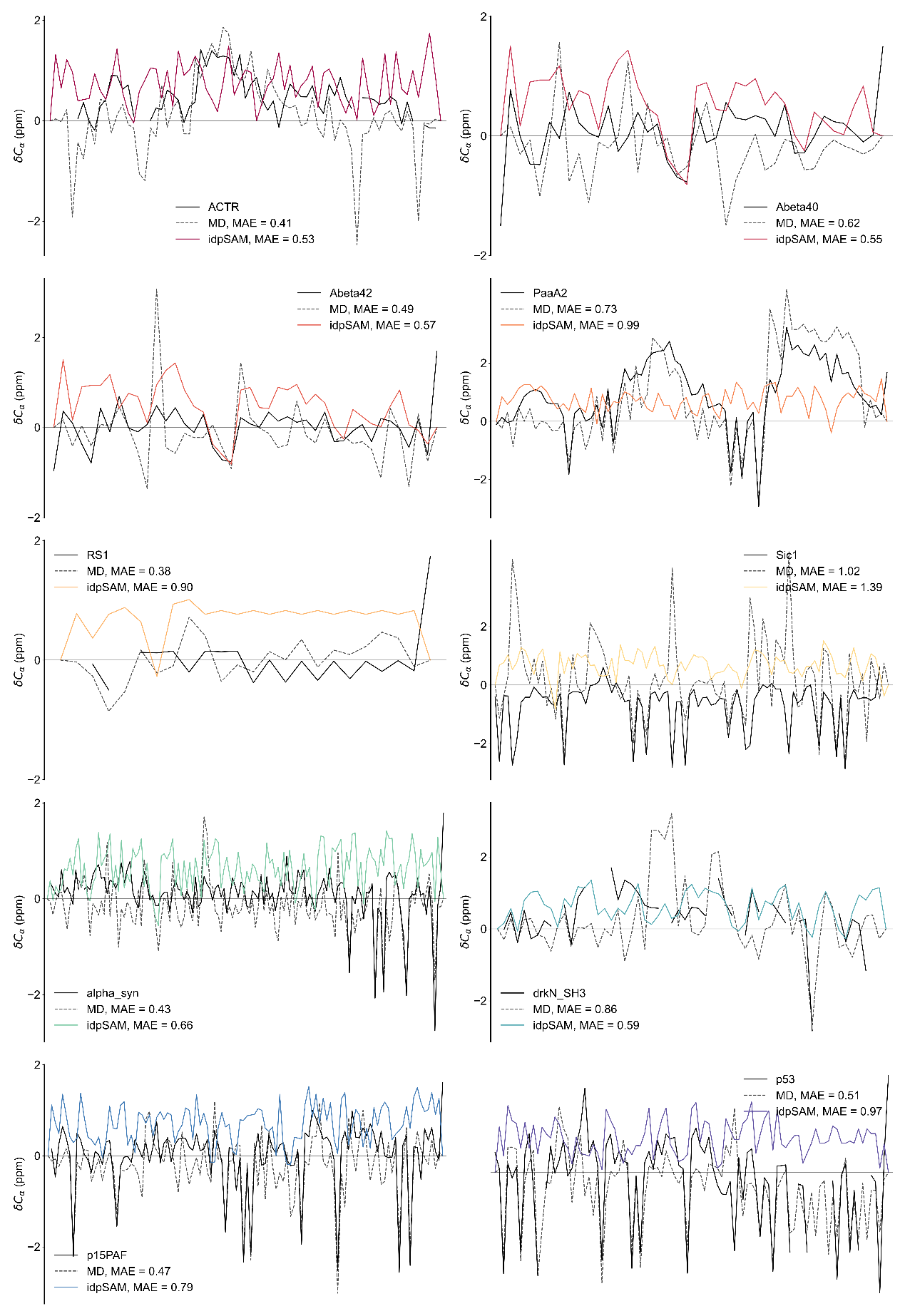


**Figure S15.** Ensemble average $C_{\alpha}$ chemical shifts of force-field-refined **idpSAM** generated conformation ensembles (colored) on 10 IDP systems. Experimental values are plotted as black solid lines.


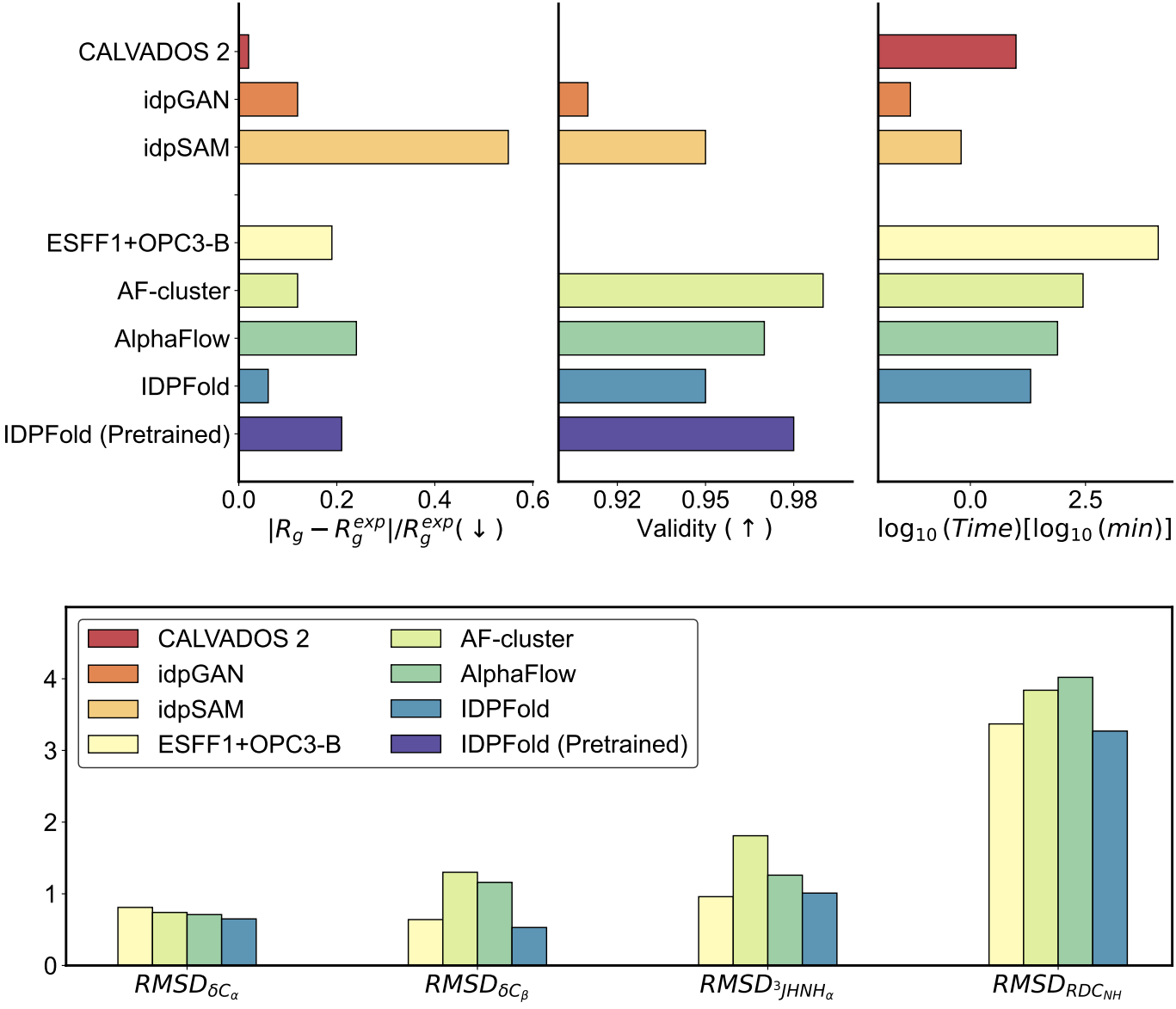


**Figure S16.** Benchmark of generative deep learning methods on IDP ensemble prediction.


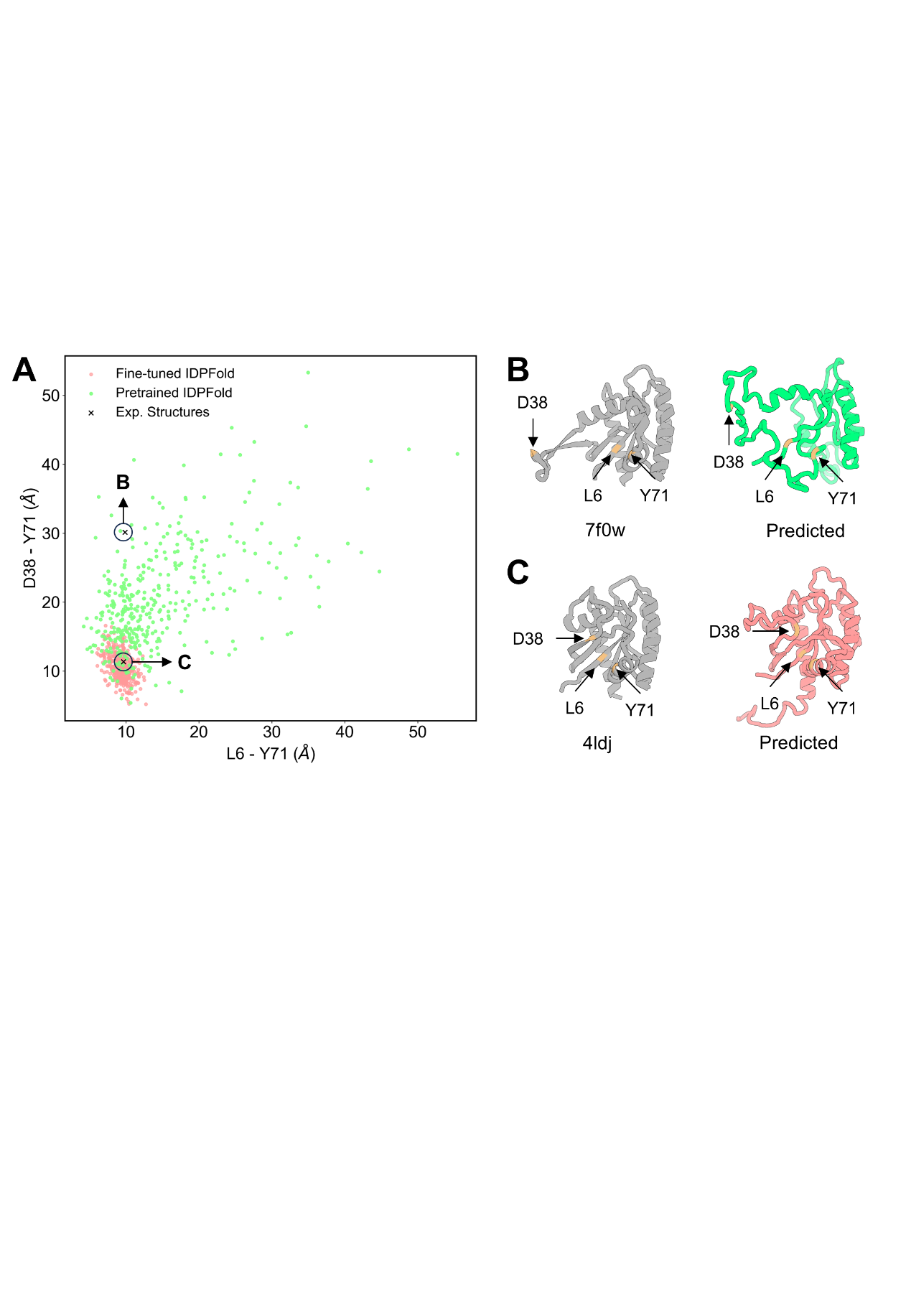


**Figure S17.** IDPFold generates a wide range of conformations for allosteric proteins. (A) IDPFold generated conformations mapped on L6-Y71-D38 distance plane. (B) Fine-tuned IDPFold generated conformation compared to X-ray structure (PDB ID: 7f0w). (C) Pretrained IDPFold generated conformation compared to X-ray structure (PDB ID: 4ldj)^34^.


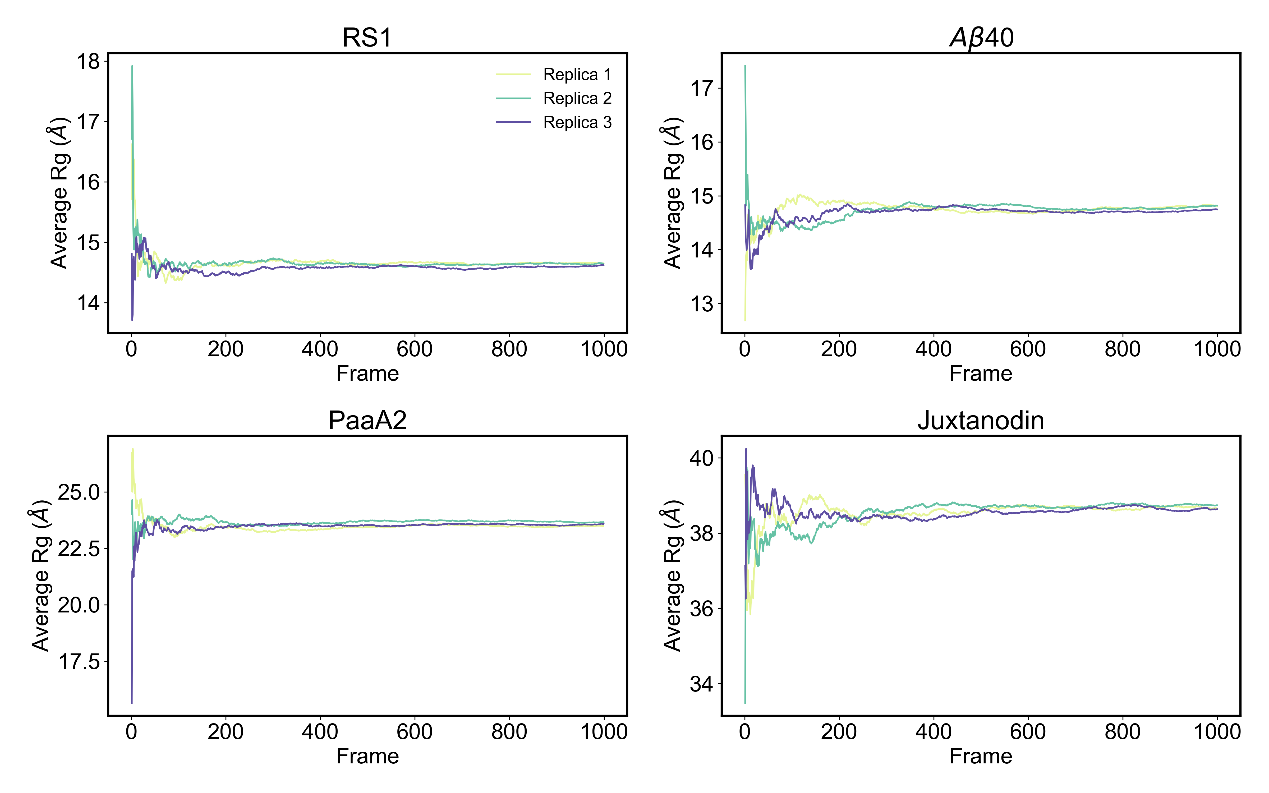


**Figure S18.** Convergence of IDPFold-generated ensembles.

### References

1. Mittag, T. *et al.* Structure/Function Implications in a Dynamic Complex of the Intrinsically Disordered Sic1 with the Cdc4 Subunit of an SCF Ubiquitin Ligase. *Structure* **18**, 494–506 (2010).

2. Granata, D. *et al.* The inverted free energy landscape of an intrinsically disordered peptide by simulations and experiments. *Sci. Rep.* **5**, 15449 (2015).

3. Hou, L. *et al.* Solution NMR Studies of the Aβ(1−40) and Aβ(1−42) Peptides Establish that the Met35 Oxidation State Affects the Mechanism of Amyloid Formation. *J. Am. Chem. Soc.* **126**, 1992–2005 (2004).

4. Roche, J., Shen, Y., Lee, J. H., Ying, J. & Bax, A. Monomeric Aβ1–40 and Aβ1–42 Peptides in Solution Adopt Very Similar Ramachandran Map Distributions That Closely Resemble Random Coil. *Biochemistry* **55**, 762–775 (2016).

5. Yan, Y., McCallum, S. A. & Wang, C. M35 Oxidation Induces Aβ40-like Structural and Dynamical Changes in Aβ42. *J. Am. Chem. Soc.* **130**, 5394–5395 (2008).

6. Ball, K. A. *et al.* Homogeneous and Heterogeneous Tertiary Structure Ensembles of Amyloid-β Peptides. *Biochemistry* **50**, 7612–7628 (2011).

7. Rauscher, S. *et al.* Structural Ensembles of Intrinsically Disordered Proteins Depend Strongly on Force Field: A Comparison to Experiment. *J. Chem. Theory Comput.* **11**, 5513–5524 (2015).

8. Xiang, S. *et al.* Phosphorylation Drives a Dynamic Switch in Serine/Arginine-Rich Proteins. *Structure* **21**, 2162–2174 (2013).

9. Cragnell, C., Durand, D., Cabane, B. & Skepö, M. Coarse-grained modeling of the intrinsically disordered protein Histatin 5 in solution: Monte Carlo simulations in combination with SAXS. *Proteins Struct. Funct. Bioinforma.* **84**, 777–791 (2016).

10. Raj, P. A., Marcus, E. & Sukumaran, D. K. Structure of human salivary histatin 5 in aqueous and nonaqueous solutions. *Biopolymers* **45**, 51–67 (1998).

11. De Biasio, A. *et al.* p15PAF Is an Intrinsically Disordered Protein with Nonrandom Structural Preferences at Sites of Interaction with Other Proteins. *Biophys. J.* **106**, 865–874 (2014).

12. Alborghetti, M. R. *et al.* Human FEZ1 Protein Forms a Disulfide Bond Mediated Dimer: Implications for Cargo Transport. *J. Proteome Res.* **9**, 4595–4603 (2010).

13. Foucault, M. *et al.* UV and X-ray structural studies of a 101-residue long Tat protein from a HIV-1 primary isolate and of its mutated, detoxified, vaccine candidate. *Proteins Struct. Funct. Bioinforma.* **78**, 1441–1456 (2010).

14. Hofmann, H. *et al.* Polymer scaling laws of unfolded and intrinsically disordered proteins quantified with single-molecule spectroscopy. *Proc. Natl. Acad. Sci.* **109**, 16155–16160 (2012).

15. Moncoq, K. *et al.* SAXS Study of the PIR Domain from the Grb14 Molecular Adaptor: A Natively Unfolded Protein with a Transient Structure Primer? *Biophys. J.* **87**, 4056–4064 (2004).

16. Boze, H. *et al.* Proline-Rich Salivary Proteins Have Extended Conformations. *Biophys. J.* **99**, 656–665 (2010).

17. Sterckx, Y. G. J. *et al.* Small-Angle X-Ray Scattering- and Nuclear Magnetic Resonance-Derived Conformational Ensemble of the Highly Flexible Antitoxin PaaA2. *Structure* **22**, 854–865 (2014).

18. Kjaergaard, M. *et al.* Temperature-dependent structural changes in intrinsically disordered proteins: Formation of α‒helices or loss of polyproline II? *Protein Sci.* **19**, 1555–1564 (2010).

19. Ebert, M.-O., Bae, S.-H., Dyson, H. J. & Wright, P. E. NMR Relaxation Study of the Complex Formed Between CBP and the Activation Domain of the Nuclear Hormone Receptor Coactivator ACTR†. *Biochemistry* **47**, 1299–1308 (2008).

20. Iešmantavičius, V. *et al.* Modulation of the Intrinsic Helix Propensity of an Intrinsically Disordered Protein Reveals Long-Range Helix–Helix Interactions. *J. Am. Chem. Soc.* **135**, 10155–10163 (2013).

21. Choy, W.-Y. *et al.* Distribution of molecular size within an unfolded state ensemble using small-angle X-ray scattering and pulse field gradient NMR techniques1. *J. Mol. Biol.* **316**, 101–112 (2002).

22. Lee, J. H. *et al.* Heterogeneous binding of the SH3 client protein to the DnaK molecular chaperone. *Proc. Natl. Acad. Sci.* **112**, E4206–E4215 (2015).

23. Ruskamo, S. *et al.* Juxtanodin is an intrinsically disordered F-actin-binding protein. *Sci. Rep.* **2**, 899 (2012).

24. Morar, A. S., Olteanu, A., Young, G. B. & Pielak, G. J. Solvent-induced collapse of α-synuclein and acid-denatured cytochrome c. *Protein Sci.* **10**, 2195–2199 (2001).

25. Structures of Two Repeats of Spectrin Suggest Models of Flexibility. *Cell* **98**, 523–535 (1999).

26. Uversky, V. N. *et al.* Biophysical Properties of the Synucleins and Their Propensities to Fibrillate: INHIBITION OF α-SYNUCLEIN ASSEMBLY BY β- AND γ-SYNUCLEINS*. *J. Biol. Chem.* **277**, 11970–11978 (2002).

27. Mylonas, E. *et al.* Domain Conformation of Tau Protein Studied by Solution Small-Angle X-ray Scattering. *Biochemistry* **47**, 10345–10353 (2008).

28. Uversky, V. N. *et al.* Natively Unfolded Human Prothymosin α Adopts Partially Folded Collapsed Conformation at Acidic pH. *Biochemistry* **38**, 15009–15016 (1999).

29. Wells, M. *et al.* Structure of tumor suppressor p53 and its intrinsically disordered N-terminal transactivation domain. *Proc. Natl. Acad. Sci.* **105**, 5762–5767 (2008).

30. Borysik, A. J., Kovacs, D., Guharoy, M. & Tompa, P. Ensemble Methods Enable a New Definition for the Solution to Gas-Phase Transfer of Intrinsically Disordered Proteins. *J. Am. Chem. Soc.* **137**, 13807–13817 (2015).

31. Chukhlieb, M., Raasakka, A., Ruskamo, S. & Kursula, P. The N-terminal cytoplasmic domain of neuregulin 1 type III is intrinsically disordered. *Amino Acids* **47**, 1567–1577 (2015).

32. Leyrat, C. *et al.* The N0-binding region of the vesicular stomatitis virus phosphoprotein is globally disordered but contains transient α-helices. *Protein Sci.* **20**, 542–556 (2011).

33. Kung, C. C.-H. *et al.* Structural analysis of poly-SUMO chain recognition by the RNF4-SIMs domain. *Biochem. J.* **462**, 53–65 (2014).

34. Hunter, J. C. *et al.* In situ selectivity profiling and crystal structure of SML-8-73-1, an active site inhibitor of oncogenic K-Ras G12C. *Proc. Natl. Acad. Sci. U. S. A.* **111**, 8895–8900 (2014).
